# Evaluation of *Rosa* germplasm resources and analysis of floral fragrance components in *R. rugosa*

**DOI:** 10.3389/fpls.2022.1026763

**Published:** 2022-10-12

**Authors:** Xi Cheng, Yan Feng, Dongliang Chen, Chang Luo, Xiaofang Yu, Conglin Huang

**Affiliations:** ^1^ Institute of Grassland, Flowers and Ecology, Beijing Academy of Agriculture and Forestry Sciences, Beijing, China; ^2^ College of Landscape Architecture, Sichuan Agricultural University, Chengdu, China

**Keywords:** *Rosa rugosa*, floral components, metabolomics, HS-SPME-GC-MS, germplasm resources

## Abstract

*Rosa rugosa* (Rosaceae) is an important functional plant used in food products, tea, and aromatherapy. Characteristics of *R. rugosa* varieties based on the biological traits and floral fragrant components were studied by applying an analytic hierarchy process, headspace solid-phase microextraction gas chromatography–mass spectrometry, and metabolomic analysis. The 77 *Rosa* accessions (comprising 27 *R. rugosa* varieties, 43 scented *R. hybrida* cultivars, and seven fragrant *R.* species) were grouped into nine classes based on 17 morphological characters and 16 targeted fragrant substances by cluster analysis. Three *R. rugosa* cultivars differing in fragrance type were selected for volatile metabolomics analysis at four stages of flower development. In total, 156 differential volatile organic compounds (VOC) were detected and the VOC content patterns were further investigated in two important metabolic pathways (the monoterpenoid biosynthetic pathway, and the phenylalanine, tyrosine, and tryptophan biosynthesis pathway). The results provide a foundation for efficient use of *Rosa* germplasm and insights into the utilization of *R. rugosa* as a functional flower.

## 1 Introduction

Rugosa rose (*Rosa rugosa*) is a deciduous shrub of the genus *Rosa* that originated in China. The flowers have important applications in medicine and the food industry. The petals are rich in a variety of amino acids and trace elements required by the human body, and are raw materials for natural flavorants, with strong potential for development and applied uses. As a result of long-term artificial and natural selection, the germplasm resources of *R. rugosa* are highly diverse. The species is native to northwestern, southwestern, and northern China and East Asia, and is cultivated worldwide but especially in Japan and South Korea. China has a long history of planting rugosa rose, which is a valuable resource and high-quality original species for the breeding of cultivated hybrid roses. Rugosa rose can be hybridized with all cultivated and wild species of *Rosa* to produce fertile offspring. Thus, it is the first choice for the production of high-quality progeny (Xu et al., 2021). The types and contents of volatile organic compounds synthesized in the flowers differ among *R. rugosa* cultivars (Dani et al., 2021). In addition, the species exhibits morphological variability, such as in the flowers, leaves, branches, and thorns, and variation in growth potential (Yang et al., 2020; Mostafavi et al., 2021). A comprehensive, systematic evaluation of *R. rugosa* cultivars is needed to ensure the most effective use of *R. rugosa* germplasm and for directional breeding of new cultivars. In the present study, we developed an analytic hierarchy process (AHP) method to comprehensively evaluate and analyze *R. rugosa* materials. The model compared traits associated with flower quality, growth vigor, and ease of harvesting among the cultivars.

Floral scent is an important characteristic of *R. rugosa* and plays major roles in the interactions of plants with other organisms (Farré-Armengol et al., 2013). The type and content of volatile organic compounds (VOCs) in flowers may be species specific and associated with the stage of flower development (Wang et al., 2014), influencing the quality of *R. rugosa* cultivars. Zhou et al. (2020a) studied the chemical profiles of volatile compounds of flower extracts at different flowering stages of rose and found that the production and release of floral VOCs from double flowers were delayed compared with those of single flowers. Rugosa rose scent contains more than 100 VOCs, consisting of terpenoids, benzene/phenyl propionic acids, fatty acid derivatives, and other chemical families such as phenol methyl ethers. Raymond et al. (2018) conducted a biochemical analysis of the main VOCs in the petals of six species of *Rosa* (*R. chinensis* ‘Old Blush’, *R. gigantea*, *R.* × *damascena*, *R. gallica*, *R. moschata*, and *R. wichurana*). Alizadeh and Fattahi (2021) detected geraniol, citral, methyl linoleate, *n*-heneicosane, and *n*-octane as the major components of essential oils in *R.* × *damascena*. Magnard et al. (2015) reported that monoterpenes comprise as much as 70% of the scent in some rose cultivars. Wang and Yao (2013) determined that the most abundant scent components detected in *R. rugosa* and *R.* × *damascena* include geraniol, phenethyl alcohol, and citronellol. Zhou et al. (2020b) discovered novel wild tea scented roses in China and evaluated various rose species using sensory and incense tone compounds.

The fragrant components of *R. rugosa* are used in perfumery, aromatherapy, and cosmetics. The study of floral metabolites and their application provides a theoretical basis for the selection and breeding of *R. rugosa* cultivars, and lays a theoretical foundation for the establishment of a rugosa rose ecological garden of edible, ornamental and medicinal value. In the present study, a targeted metabolomic analysis of 27 *R. rugosa* cultivars, 43 scented *R. hybrida* cultivars, and seven fragrant *Rosa* species in the flowering period was conducted. The latter fragrant cultivars and species were compared with *R. rugosa* as exogenous germplasm. Based on the metabolomic analysis, the floral fragrant components were detected at four stages of flower development in three *R. rugosa* cultivars that differed in fragrance type. Multivariate statistical analysis was used to accurately identify differential metabolites and metabolic pathways. Based on the phenotypic and metabolite data, we performed a more detailed analysis of three *R. rugosa* cultivars selected for their high economic value and suitability for landscaping and industrial applications. The crucial floral metabolites and the associated secondary metabolic pathways were identified. The ultimate objective of this study was to provide a reference for further screening of genes associated with fragrance in *R. rugosa*, and to lay a foundation for genetic engineering of floral metabolites and the breeding of new rose cultivars.

## 2 Materials and methods

### 2.1 Plant material

**Figure 1:**
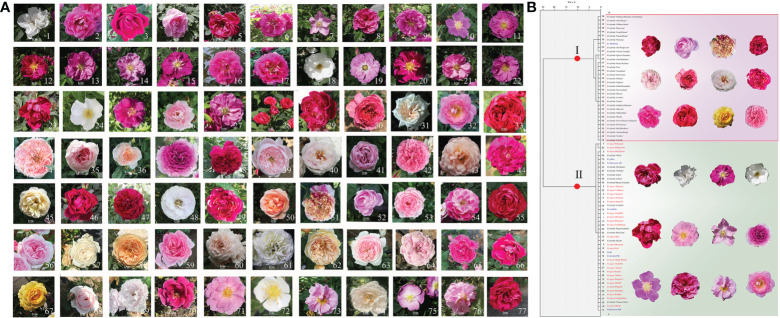
Study materials comprising 27 *Rosa rugosa* cultivars, 43 scented *R. hybrida* cultivars, and seven fragrant *R.* species, and cluster analysis of morphological characters in *Rosa* accessions. **(A)** 1–27, *R. rugosa* cultivars; 28–70, scented *R. hybrida* cultivars; 71, *R.* × *damascena*; 72, *R.* spp.; 73, *R. gallica*; 74, *R.* × *damascena* ‘Alba’; 75, *R. davurica*; 76, *R. centifolia*; 77, *R.* ‘Dianhong’. **(B)** Dendrogram based on similarity in morphological traits. *Rosa rugosa* accessions are in red font, scented *R. hybrida* cultivars are in black font, and scented *Rosa* species are in blue font. The internal bar is 1 cm.

We collected material of 27 *R. rugosa* cultivars, 43 scented *R. hybrida* cultivars, and seven fragrant *Rosa* species for biological trait investigation. We analyzed nine annual flowering branches of each plant from nine different individuals, which were considered as biological replicates in the same environment, and froze petals from the sampled branches for targeted metabolomics analysis (**Figure 1A** and **Table S1**). Three biological replicates were collected and detected from three individual plants of each variety. The materials planted in the *R. rugosa* germplasm resource nursery of the Beijing Academy of Agriculture and Forestry Sciences at Beilangzhong, Zhaoquanying, Shunyi, Beijing, China. Petals and flowering branches were sampled from May to July 2020. The *R. rugosa* cultivars ‘Hanxiang’, ‘BaiZiZhi’, and ‘Guo’ were sampled at four flowering stages (**Figure 4A**) for volatile metabolomic analysis, for which we also collected three biological replicates.

### 2.2 Methods for biological trait assessment

In accordance with the Chinese standard ‘Guidelines for the conduct of tests for distinctness, uniformity and stability—Rose (*Rosa* L.)’ (LY/T 1868-2010), we recorded the following 17 biological traits (**Table S2**): single/double petals, flower petal number, flower diameter, number of branches, plant morphology, pedicel length, internode length, crown width, main stem thickness, flowering branch thickness, plant height, total number of thorns, shape of the lower part of the thorns, and presence/absence of thorns and bristles on flowering branches and pedicels.

### 2.3 Gas chromatography–mass spectrometry

Fresh petal samples were collected and weighed, frozen in liquid nitrogen, and stored at −80°C for later analysis. We ground the sample to powder in liquid nitrogen and transferred it to a 20 mL headspace vial (Agilent, Palo Alto, CA, USA) containing saturated NaCl solution to inhibit any enzyme reaction. We sealed the vial using crimp-top caps with TFE–silicone headspace septa (Agilent). The vial was incubated at 60°C for 10 min. Headspace solid-phase microextraction (HS-SPME) was conducted by exposing the headspace of the sample for 20 min at 60°C to 65 µm divinylbenzene/carboxen/polydimethylsiloxane fiber (Supelco, Bellefonte, PA, USA). After sampling, desorption of the VOCs from the fiber coating was conducted in the injection port of the gas chromatograph (GC) apparatus (Model 8890; Agilent) at 250°C for 5 min in the splitless mode. The identification and quantification of VOCs were performed using a Model 8890 GC (Agilent) and a 5977B mass spectrometer (MS; Agilent), equipped with a 30 m × 0.25 mm × 0.25 µm DB-5MS (5% phenyl-polymethylsiloxane) capillary column. The carrier gas was helium with a linear velocity of 1.0 ml/min. The injector temperature was kept at 250°C and the detector temperature at 280°C. The oven temperature was programmed from 40°C (5 min), increasing at 5°C/min to 280°C, and held for 5 min. We recorded the mass spectra in the electron impact (EI) ionization mode at 70 eV. The quadrupole mass detector, ion source, and transfer line temperatures were set, respectively, at 150, 230, and 280°C. Mass spectra were scanned in the range *m*/*z* 30–350 amu at 1 s intervals. Identification of volatile compounds was achieved by comparing the mass spectra with the data system library (MWGC) and linear retention index.

### 2.4 Statistical analysis of targeted metabolomics data

We detected contents of citronellol, phenethyl alcohol, farnesol, nerol, and rose oxide in the petals by HS-SPME coupled to GC-MS for targeted metabolomic analysis. The procedure followed previously described methods (Zhou et al., 2015; Yun et al., 2016; Raymond et al., 2018; Yuan et al., 2018), and the measured metabolites were sorted according to their content and concentration. Following the provisions of the International Standard for Oil of Rose (ISO 9843: 2003), we compared the most important metabolites with previous data on the floral components of the 16 target metabolites screened in this study comprising eight terpenes (α-farnesene, nerolidol, nerol, farnesol, rose ether, citronellal, citronellol, and linalool), four esters (neryl acetate, citronellyl acetate, phenethyl acetate, and geranyl acetate), two phenols (eugenol and methyleugenol), one alcohol (phenethyl alcohol), and one heterocyclic compound (*trans*-linalool oxide). We prepared standard curves for the 16 target substances and quantified their contents. The data for the target metabolites of the 27 *R. rugosa* cultivars were visualized in histograms and phenograms.

### 2.5 Comparative analysis of traits of *R. rugosa*, fragrant *R. hybrida*, and scented *Rosa* species

The data for the 27 *R. rugosa* accessions and other *Rosa* materials were analyzed using Toolbox for Biologists v1.068 software of Chen et al. (2020). We constructed a heatmap to display the 18 traits (including morphological characters and fragrant substances) for the *Rosa* species, where different colors indicate differences in traits among materials. We used IBM SPSS Statistics 20 (IBM Corporation, Armonk, NY, USA) software for analysis to intuitively reflect the classification of *Rosa* germplasm resources as a phenogram. The main characters were clustered based on Euclidean distances using Ward’s (sums of squared deviations) method.

### 2.6 Comprehensive evaluation of *R. rugosa* cultivars

To generate an analytic hierarchy process (AHP) model, we first consulted 15 experts on ornamental flowering plants and provided each with a questionnaire to determine which traits should be evaluated. Experts were asked to select a value of 1–9 for pairwise comparisons of characters, where 1 indicated equal importance, 3 indicated slightly favored, 5 indicated strongly favored, and 7 indicated very strongly favored. According to the relative importance of each element in the upper layer, the relative importance of any two elements in the same layer to the upper layer was assigned a weight to form a paired comparison matrix. This procedure established which of the two factors was more important and the degree of importance. The questionnaire responses were used to calculate the values in the judgment matrix. The consistency of each judgment matrix was tested (a consistency ratio [CR] < 0.1 passed the consistency test) and the weight was determined.

We constructed the *R. rugosa* AHP model (**Figure 3A**), which was divided into three layers. The left (decision-making) layer was the resource application evaluation of *R. rugosa* (‘An industry application evaluation’ layer, leftmost in **Figure 3A**). The middle (criteria) layer was flower quality evaluation, plant growth and vigor, and ease of harvesting (‘B1 Quality trait evaluation’, ‘B2 Vigor status evaluation’, and ‘B3 Harvest difficulty evaluation’). The right (index) layer was the investigated biological traits and floral metabolite contents (22 characters designated F1–F22). To conduct calculations, the model was imported into the YAAHP software (standard version; Shanxi Yuanju Software Technology Co., Ltd., Shanxi, China). The actual score of each element of the index layer was the statistical value for that trait, which was input into the software.

On the basis of actual measurements and field observations, we established the scoring standard for *R. rugosa*. We scored the field survey data. The final score for each cultivar was obtained by multiplying the weight for each evaluation element by the field score.

### 2.7 Statistical analysis of volatile metabolomics data

We used Mass Hunter software (Agilent) to extract and analyze the original data for volatile metabolites obtained by GC-MS, and then analyzed the data qualitatively, quantitatively, and statistically. We prepared the quality control (QC Mix) sample by combining the extracts of all samples to analyze the repeatability of the results. The contents of volatile metabolites at four stages of flower development for three *R. rugosa* cultivars were compressed into *n* principal components to describe the characteristics of the original data set. We analyzed 13 groups of samples (including the QC Mix), and analyzed the differences between and within groups of volatile metabolite data samples by principal component analysis. The floral metabolite content of three *R. rugosa* cultivars in different periods were processed by unit variance scaling, and a heatmap was generated using the ‘pheatmap’ R package. An orthogonal partial least squares discriminant analysis (OPLS-DA) combines the orthogonal signal correction and PLS-DA methods. Twelve groups of samples were divided into nine control groups: BZ1 *vs* BZ3, BZ3 *vs* BZ5, BZ5 *vs* BZ7, GM1 *vs* GM3, GM3 *vs* GM5, GM5 *vs* GM7, HX1 *vs* HX3, HX3 *vs* HX5, and HX5 *vs* HX7. We generated a score plot (S-plot of OPLS-DA) for each group and calculated the variable importance in projection (VIP) value. Metabolites were selected that differed between samples within a group. Volcano plots were constructed to identify differential metabolites. We standardized the relative content of differential metabolites and clustered them using *K*-means clustering to analyze trends in variation.

We used the Kyoto Encyclopedia of Genes and Genomes (KEGG) database to examine metabolic networks. By comparing the metabolite composition at four stages of flower development in three *R. rugosa* cultivars, the significant differential metabolites were screened and enriched biosynthetic pathways were explored.

## 3 Results

### 3.1 Comparative analysis of traits of *R. rugosa*, fragrant *R. hybrida*, and scented *R.* species

Based on the original field survey data, a horizontal comparison was performed. The raw field data for *R. rugosa*, fragrant *R. hybrida*, and other *Rosa* species are provided in **Table S3**, the names of the fragrant *R. hybrida* cultivars and other *Rosa* species are listed in **Table S1**, and illustrations are shown in **Figure 1A**. A heatmap (**Figure S6**) was constructed to display the distribution of morphological characters and fragrant substances among the *Rosa* materials. Although *Rosa* taxa are closely related, they can be morphologically quite distinct. We detected clear differences in morphology among the studied germplasm. The number of petals of scented *R. hybrida* was generally double that of *R. rugosa* and the flower diameter was slightly larger. The peduncle of *R. hybrida* was approximately 2 cm longer than that of *R. rugosa.* The internodes of *R. rugosa* and the scented *Rosa* species were shorter than those of *R. hybrida*. *Rosa rugosa* stems carried more thorns than the other *Rosa* materials, and the thorn shape was straight and oblique. The scented *Rosa* species were more similar to *R. rugosa* in overall morphological traits.

The 77 *Rosa* accessions (*R. rugosa*, *R. hybrida*, and scented *R.* species) were divided into two distinct groups based on the morphological characters (**Figure 1B**). Thirty-three cultivars were clustered in group I, comprising exclusively *R. hybrida* cultivars except for *R.* ‘Dianhong’, and this group was further subdivided into three subgroups. Thus, the phenotype of *Rosa* ‘Dianhong’ in the field was more similar to that of *R. hybrida*. The overall traits of group I (compared with those of *R. rugosa*) were greater number of petals, broader flower diameter, and longer pedicel. The general pattern of floral diversity in *R. hybrida* is relatively rich, with diversity in petal color and proliferation of petals, which are favorable traits. Group II comprised 44 cultivars that were further divided into two subgroups. *Rosa rugosa* ‘Peking Red’ and *R. rugosa* ‘Peking White’ formed Subgroup II-1; these two accessions are very similar in floral shape, but differ in petal color. Subgroup II-2 contained all remaining *R. rugosa* accessions, scented *Rosa* species, and a number of *R. hybrida* cultivars. Thus, *R. hybrida* cultivars were largely clustered in group I, whereas *R. rugosa* and scented *Rosa* species were clustered in group II, reflecting a stronger similarity in phenotype.

### 3.2 Targeted metabolomics analysis in *R. rugosa*

**Figure 2:**
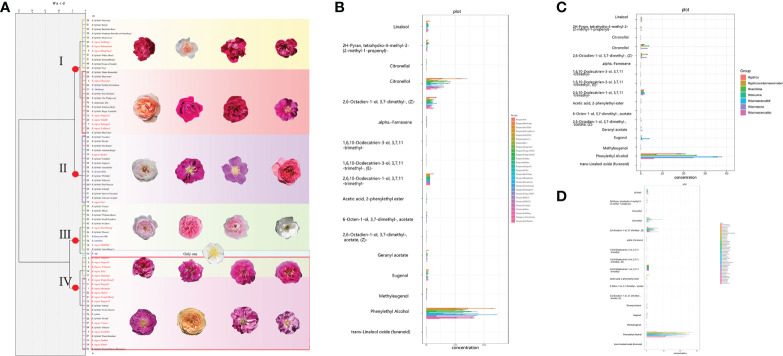
Cluster analysis of morphological characters and histogram of target metabolites in *Rosa* accessions. The unit for the floral scents is μg/g. **(A)** Dendrogram based on similarity in morphological characters. **(B)** Histogram of target metabolites in 27 *R. rugosa* cultivars. **(C)** Histogram of target metabolites in 43 fragrant *R. hybrida* cultivars. **(D)** Histogram of target metabolites in seven fragrant *Rosa* species.

Among the 16 substances of interest (**Figure 2B** and **Table S4**), the phenethyl alcohol content in all *R. rugosa* cultivars was generally high, and was substantially higher than that of the other fragrant substances (mean 11.76 µg/g). The next most abundant fragrant compounds were citronellol, nerol, and farnesol with average contents of 3.76, 1.77, and 1.27 µg/g. The contents of nerolidol, farnesol, neryl acetate, citronellal, geranyl acetate, and *trans*-linalool oxide in *R. rugosa* ‘Guo’ were the highest. The contents of nerolidol and farnesol were much higher than the other substances. Phenethyl alcohol was undetected (0 µg/g), the content of phenylethyl acetate was as low as 0.0001 µg/g, and the citronellol content was significantly lower than that of the other cultivars. *Rosa rugosa* ‘Hanxiang’ had the highest contents of citronellol and linalool, whereas the nerolidol, farnesol, α-farnesene, phenylethyl acetate, and eugenol contents were lower than the corresponding average for all cultivars. The content of methyleugenol in *R. rugosa* ‘BaiZiZhi’ was the highest among the tested *R. rugosa* cultivars, and the phenethyl alcohol content was second only to that of ‘TianEHuang’. In contrast, the contents of nerolidol, farnesol, neryl acetate, citronellal, geranyl acetate, linalool, nerol, rose oxide, and phenethyl acetate were all very low and below the overall average. The *R. rugosa* cultivars with favorable contents of fragrant substances comprised ‘Pingyin12’, ‘Pingyin8’, ‘Xihu II’, ‘FanHua’, ‘Pingyin11’, ‘DaGuo’, ‘Purple Branch’, ‘Mici’, and ‘TianEHuang’.

Among fragrant compounds in *R.* species, phenethyl alcohol showed the highest content, followed by citronellol, nerol, and farnesol (**Figures 2B–D**). The contents of fragrant components in *R. rugosa* were higher than those of the other *Rosa* materials, and was especially favorable for important substances, such as rose oxide, citronellol, citronellyl acetate, and citronellal. Compared with *R. rugosa*, fragrant *R. hybrida* cultivars had lower contents of or lacked important fragrant substances*. Rosa* × *damascena*, *R. hybrida* ‘Aunt Margy’s’, *R. hybrida* ‘Dream of Garden’, *R. hybrida* ‘Yumao’, and *R. hybrida* ‘My Beauty’ had a greater number of fragrant substances, whereas *R. hybrida* ‘Accademia’, *R. hybrida* ‘Kayla’, *R. hybrida* ‘Paul Neyron’, and *R. hybrida* ‘Neixiang’ had fewer fragrant substances. Among the seven fragrant *Rosa* species, the contents of all fragrant substances in *R. davurica* were very low and the content of five substances was 0 µg/g. The overall composition of fragrance components in *R.* ‘Dianhong’ and *R.* × *damascena* ‘Alba’ was poor.

With consideration of the 16 targeted fragrant substances, the 77 *Rosa* accessions were divided into four groups (**Figure 2A**). Twenty-seven accessions were clustered in group I, which was subdivided into two subgroups. Twelve accessions were clustered in group I-1 (nine fragrant *R. hybrida* cultivars and three *R. rugosa* cultivars) and 15 accessions were clustered in group I-2 (five *R. rugosa* cultivars, eight fragrant *R. hybrida* cultivars, and two fragrant *Rosa* species). Sixteen cultivars formed group II (with no subgroup), comprising two *R. rugosa* cultivars and 14 fragrant *R. hybrida* cultivars. Group III comprised 12 accessions in two subgroups; two *R. rugosa* cultivars, seven fragrant *R. hybrida* cultivars, and two fragrant *Rosa* species were clustered in subgroup III-1, whereas subgroup III-2 comprised a single accession (*R.* sp.). Twenty-two accessions were clustered in group IV, of which 15 were *R. rugosa* cultivars (five in group IV-1 and 10 in group IV-2). These results showed that seven fragrant *R. hybrida* cultivars, such as *R. hybrida* ‘Sweet Chariot’ and *R. hybrida* ‘Poetry Kordana’, and *R. gallica* were more similar to *R. rugosa* cultivars in fragrance components.

The contents of citronellol, phenethyl alcohol, farnesol, nerol, and rose oxide, which are important fragrance components in the *Rosa* materials, were significantly higher in the *R. rugosa* accessions than in the *R. hybrida* cultivars and scented *Rosa* species.

### 3.3 Industry application evaluation for *R. rugosa* with the AHP model

**Figure 3:**
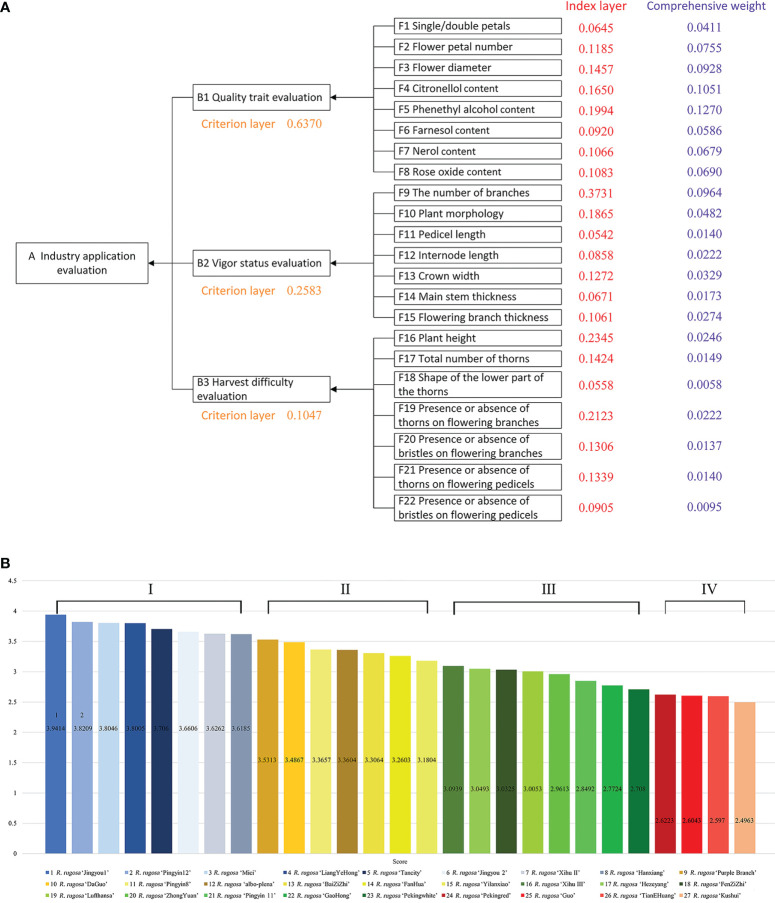
Analytic hierarchy process (AHP) model constructed for *R. rugosa* cultivars and the final scores. **(A)** AHP model constructed for *R. rugosa* cultivars. The orange numbers are values of the criterion layer (B1, B2, and B3). The red numbers are values for the index layer (F1–F22). The violet numbers are values for the comprehensive weight. **(B)** The final scores of 27 *R. rugosa* cultivars.

Based on 22 evaluation characters, we calculated the weighted scores for each of 27 test cultivars with the AHP and screened the superior cultivars. Seventeen biological traits (**Table S2** for the raw field data) and five fragrant metabolites (the metabolite contents are listed in **Table S5** and examples of detected integral graphs are shown in **Figures S1**-**S5**) were used as factors for evaluation of industrial applications for *R. rugosa.* Fifteen questionnaires were distributed to authoritative experts. The consistency of responses in the returned questionnaires was found to be valid and the questionnaire results were combined. In the ‘Industry application evaluation’ (A), quality trait evaluation (B1) was the most important factor in quality control (63.7%, the orange numbers in **Figure 3A**), followed by vigor status evaluation (B2; 25.83%) and harvest difficulty evaluation (B3; 10.47%). Because the size, number, and shape of thorns greatly affects the ease of harvesting, thorniness affects labor costs and working hours in the industry (**Figure 3A**).

After calculating the weight value (*F*_i_) of each specific character relative to the attribute, the weight of each character relative to the total comprehensive evaluation was calculated. The weighted sorting weight value of the indicator layer “F1” relative to the decision layer “a” was equal to the product of the sorting weight value *W*_i_ (0.0645, the red number in **Figure 3A** **)** of “F1” relative to “B1” and the sorting weight value *W*_i_ (0.6370) of “B1” relative to “a” (0.0645 × 0.6370 = 0.0411, the violet number in **Figure 3A**). With regard to industrial applications, phenethyl alcohol (F5), citronellol (F4), branch number (F9), and flower diameter (F3) received the highest weight coefficients.

Based on the statistical data for the biological traits and the detection of floral metabolites, **Table S6** shows the standard of evaluation index scores. **Figure 3B** shows the final weighted scores and grading of each element in the index layer calculated with respect to the decision-making layer. The *R. rugosa* materials were assigned to four grades on the basis of their weighted scores: Grade I, weighted score ≥ 3.6; Grade II, weighted score 3.1–3.6; Grade III, weighted score 2.7–3.1; and Grade IV, weighted score < 2.7.

### 3.4 Metabolomic analysis of volatile organic compounds of three *R. rugosa* cultivars with different fragrance types

The volatile organic component metabolites of three *R. rugosa* cultivars differing in fragrance type at four stages of flower opening were analyzed and a total ion current diagram was generated (**Figure S7**). In total, 174 substances were identified, including 63 (36%) terpenoids, 10 (6%) aldehydes, 10 (6%) alcohols, 16 (9%) lipids, 7 (4%) phenols, 12 (7%) ketones, 18 (10%) alkanes, 16 (10%) aromatics, and 21 (12%) other substances (**Table 1**).

**Table 1:** Volatile organic metabolites detected in three *Rosa rugosa* cultivars with different fragrance types.

Substances	Type	Concentration (µg/L)	Proportion
Monoterpenes	63	1.18E+09	40.97%
Esters	16	9.51E+07	3.29%
Heterocyclic compounds	10	1.59E+08	5.50%
Alkene	5	1.10E+07	0.38%
Alkane	18	3.11E+07	1.07%
Ketones	12	2.98E+08	10.29%
Acids	2	8.14E+06	0.28%
Aldehydes	10	1.14E+08	3.96%
Phenols	7	1.76E+08	6.07%
Aromatic hydrocarbons	17	5.27E+07	1.82%
Alcohols	10	7.46E+08	25.79%
Amines	1	5.48E+05	0.02%
Others	2	1.54E+07	0.53%
Sulfides	1	5.38E+05	0.02%
Total	174	2112383197	100%

#### 3.4.1 Principal component analysis

Principal component analysis was conducted on the original metabolite data for the three *R. rugosa* cultivars (**Figure S8**). The CR of PC1 was 32.17% and that of PC2 was 16.62% (a combined CR of 48.79%). The principal components explained the metabolite characteristics of the samples. Differences between the sample groups were greater than those within the sample group. Significant differences were observed in the volatile metabolites detected at each developmental stage among the three *R. rugosa* cultivars. The QC Mix (all samples mixed) was placed centrally in the scatterplot, which indicated that the mixing was satisfactory and that analysis of the samples under the same treatment method showed good repeatability.

#### 3.4.2 Cluster analysis

**Figure 4:**
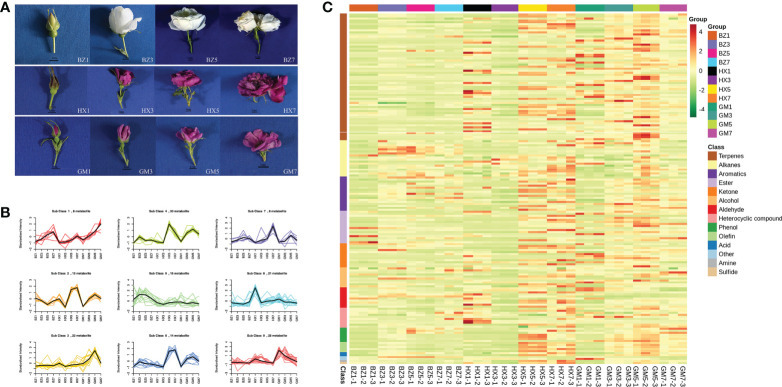
Flower development stages, *K*-means diagram, and heatmap of volatile metabolites in three *R. rugosa* cultivars. The unit for the floral scents is μg/g. **(A)** Stages of flower development for each *R. rugosa* cultivar. BZ1, flower-bud stage of ‘BaiZiZhi’, BZ2, early-blooming stage of ‘BaiZiZhi’, BZ3, full-blooming stage of ‘BaiZiZhi’, BZ4, withering stage of ‘BaiZiZhi’; HX1, flower-bud stage of ‘Hanxiang’, HX2, early-blooming stage of ‘Hanxiang’, HX3, full-blooming stage of ‘Hanxiang’, HX4, withering stage of ‘Hanxiang’; GM1, flower-bud stage of ‘Guo’, GM2, early-blooming stage of ‘Guo’, GM3, full-blooming stage of ‘Guo’, GM4, withering stage of ‘Guo’. The internal bar is 1 cm. **(B)** Relative contents of standardized differential metabolites in each cultivar. Subclass represents the category number of metabolites with the same change trend. **(C)** Heatmap of the metabolites in each *R. rugosa* cultivar.

The metabolite content of *R. rugosa* ‘Hanxiang’ flowers was distinctly concentrated in the full-blooming stage and the withering stage (HX5 and HX7) (**Figure 4C**). With the progression of flowering, terpenes predominated at the full-blooming stage and withering stage, followed by alkanes, aromatics, and esters. The metabolite content of *R. rugosa* ‘Guo’ was highest in the full-blooming stage (GM5) and the content gradually increased with the progression of flowering. In *R. rugosa* ‘BaiZiZhi’ the highest metabolite contents were at the full-blooming stage (BZ5). The contents of volatile substances in ‘BaiZiZhi’ at each flowering stage were significantly lower than those of ‘Guo’ and ‘Hanxiang’. Relative contents of flower fragrance substances increased gradually during flower development and peaked at the full-blooming stage.

#### 3.4.3 Screening and content trend analysis of differential metabolites in *R. rugosa*

Nine groups of PLS-DA models were established and the S-plot represented the CR of each metabolite to the group (**Figure S9**). After PLS-DA analysis, significant differential metabolites were selected according to the following criteria: VIP > 1, up-regulated metabolite fold change ≥ 2, and down-regulated metabolite fold change ≤ 0.5. The numbers of differential metabolites detected in the comparisons BZ1 *vs*. BZ3, BZ3 *vs*. BZ5, and BZ5 *vs*. BZ7 were 86, 56, and 19, respectively; those in the comparisons GM1 *vs*. GM3, GM3 *vs*. GM5, and GM5 *vs*. GM7 were 87, 30, and 39, respectively; and those in the comparisons HX1 *vs*. HX3, HX3 *vs*. HX5, and HX5 *vs*. HX7 were 62, 66, and 30, respectively. The total number of differential metabolites in the nine groups was 156 (**Figure S10**).

The general trend for total differential metabolites is shown in **Figure 4B**. The change trend of the 156 substances was nine (Classes 1–9). Among the three cultivars, the contents of eight metabolites (e.g., 1,3-cyclohexadiene-1-carboxaldehyde, 2,6,6-trimethyl-,eugenol, and 2,6-octadien-1-ol,3,7-dimethyl-, acetate, (Z)-) in Class 1 showed a gradual upward trend from the flower-bud stage to the withering stage. The contents of 25 metabolites (e.g., tridecane, methyl salicylate, and hexanal) in Class 9 decreased gradually from the flower-bud stage to the withering stage. Thirty metabolites (e.g., linalool, geraniol, and α-thujene) were grouped in Class 4. Eight metabolites (e.g., (2S,4R)-4-methyl-2-(2-methylprop-1-en-1-yl) tetrahydro-2H-pyran, benzene, 1,2,3-trimethoxy-5-(2-propenyl)-, and methyleugenol) were in Class 7. Twenty-seven metabolites (e.g., L-α-terpineol, 6-octen-1-ol,3,7-dimethyl-,formate, citronellal, and citronellol) were placed in Classes 2 and 6. Twenty-two metabolites (e.g., 1,6,10-dodecatrien-3-ol,3,7,11-trimethyl-,(E)-, *trans*-linalool oxide (furanoid), α-farnesene, and geranyl acetate) were grouped in Class 3. Fifteen metabolites (e.g., phenylethyl alcohol, styrene, pentadecane, and hexadecane) were included in Class 5.

In summary, among the three cultivars, the highest total relative content of differential metabolites was detected in ‘Hanxiang’, followed by ‘Guo’, and the lowest was that of ‘BaiZiZhi’. The relative contents of most fragrant substances (e.g., nerolidol, α-farnesene, geranyl acetate, citronellal, citronellol, and methyleugenol) of ‘Hanxiang’ were much higher than those of the other two cultivars, of which ‘Guo’ showed the second-highest contents. ‘Guo’ had the highest relative contents of methyl salicylate, 2,6-octadien-1-ol,3,7-dimethyl-,acetate,(Z)-, hexanal, and eugenol. Although ‘BaiZiZhi’ compared poorly with regard to these substances, it had the highest content of 2-phenethyl alcohol, and the contents of aromatic compounds, such as styrene, *p*-xylene, and benzeneacetaldehyde, was high (aromatic compounds). The differential metabolites data are provided in **Table S7**.

#### 3.4.4 Enrichment of metabolite pathways in *R. rugosa*

Based on the preceding results, we screened the differential metabolites for enriched KEGG pathways. Important metabolic pathways included sesquiterpene and triterpene biosynthesis, phenylpropane biosynthesis, monoterpene biosynthesis and metabolism, and secondary metabolite biosynthesis (**Figure S11**). In comparisons for ‘BaiZiZhi’, differential metabolites in BZ1 *vs*. BZ3 were most highly enriched in sesquiterpene and triterpene biosynthesis. Valens and (+)-δ-carbene were down-regulated and α-farnesene was up-regulated (ko00909). The differential metabolites in BZ3 *vs*. BZ5 were most distinctly enriched in monoterpene biosynthesis; geraniol and L-α-terpineol were down-regulated (ko00902). The differential metabolites in BZ5 *vs*. BZ7 were highly enriched in sesquiterpene and triterpene biosynthesis, with only one terpene (α-farnesene), which was down-regulated (ko00909). In the comparisons for ‘Guo’, the differential metabolites in GM1 *vs*. GM3 were most highly enriched in monoterpene biosynthesis; geraniol and (1s)-6,6-dimethyl-2-methylene-bicycloheptane were up-regulated (ko00902). Among the differential metabolites of GM3 *vs*. GM5, the most distinct enrichment was in terpene biosynthesis; 3,7,11-trimethyl-2,6,10-dodecyl was up-regulated (ko00900). The differential metabolites in GM5 *vs*. GM7 were most highly enriched in phenylpropane biosynthesis; eugenol was up-regulated and methyleugenol was down-regulated (ko00940). In comparisons for ‘Hanxiang’, differential metabolites in HZ1 *vs*. HZ3 were most highly enriched in sesquiterpene and triterpene biosynthesis; valens, (−)-β-diterpenes, and α-farnesene were down-regulated (ko00909). Differential metabolites of HZ3 *vs*. HZ5 were most obviously enriched in monoterpene biosynthesis; geraniol, (1s)-6,6-dimethyl-2-methylene-bicycloheptane, and L-α-terpineol were up-regulated (ko00902).

#### 3.4.5 Analysis of metabolite pathways in *R. rugosa*

**Figure 5:**
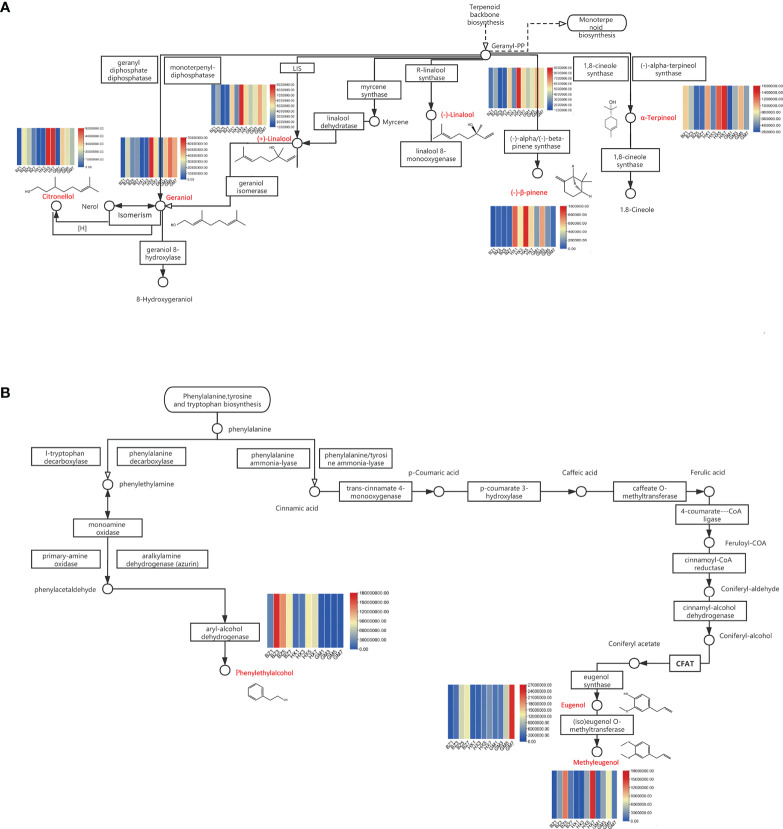
Enriched biosynthetic pathways in *Rosa rugosa*. The patterns in volatile organic compound (VOC) contents over the four flowering stages for three varieties of *R. rugosa* are provided above or below the differential metabolites (red font). **(A)** Monoterpenoid biosynthetic pathway. **(B)** Phenylalanine, tyrosine, and tryptophan biosynthetic pathway.

We screened the differential metabolites that were shared in the two most important enriched pathways: the monoterpenoid biosynthetic pathway, and the phenylalanine, tyrosine, and tryptophan biosynthesis pathway. The monoterpenoid biosynthetic pathway includes many important fragrant substances, such as citronellol, nerol, geraniol, linalool, myrcene, α-terpineol, and (−)-β-pipene (Feng et al., 2014). The synthesis of these substances can be clearly seen in the biosynthetic pathways diagram (**Figure 5A**). A heatmap showed that the synthesis of these compounds coincided with the progression of flower development (**Figure 5A**). The contents of citronellol, geraniol, and linalool peaked at the early-blooming stage (BZ3) in ‘BaiZiZhi’ and the full-blooming stage (HZ5) of ‘Hanxiang’. The content of (−)-β-pipene in ‘BaiZiZhi’ showed no obvious change during flower development, but α-terpineol content was highest in the flower-bud stage of ‘BaiZiZhi’ (BZ1). The content of (−)-β-pipene in ‘Hanxiang’ was highest in the full-blooming stage (HZ5), and that of α-terpineol was highest in the withering stage (HZ7). At the full-blooming stage of ‘Guo’ (GM5), geraniol, linalool, and α-terpineol showed the highest contents. Citronellol and (−)-β-pipene contents were highest in the early-blooming stage (GM3). Linalool is catalyzed by geraniol isomerase to yield geraniol. The relative content of linalool in ‘BaiZiZhi’ was low in the early-blooming stage (BZ3), whereas the relative content of geraniol in the early-blooming stage (BZ3) increased significantly. This result indicated that the catalytic reaction between linalool and geraniol isomerase starts from the early-blooming stage (BZ3) and continues to the full-blooming stage (BZ5) and withering stage (BZ7). The relative content of geraniol in ‘Guo’ at the full-blooming stage (GM5) was significantly higher than that of linalool, indicating that the catalytic reaction between linalool and geraniol isomerase occurred in the full-blooming stage (GM5).

Biosynthetic pathways for phenylalanine, tyrosine, and tryptophan are extremely common in plants. These include the pathways for phenethyl alcohol and eugenol biosynthesis. These biosynthetic pathways include the synthesis of phenethyl alcohol, eugenol, methyleugenol, phenylalanine, and isoeugenol (**Figure 5B**). A heatmap indicated that the phenethyl alcohol content of ‘BaiZiZhi’ was highest in the early-blooming stage (BZ3) and that of ‘Hanxiang’ in the full-blooming stage (HX5), whereas phenethyl alcohol was not detected in ‘Guo’. The eugenol content was highest in the withering stage (BZ7 and GM7) of ‘BaiZiZhi’ and ‘Guo’, and also was high in the full-blooming stage (HX5) of ‘Hanxiang’. The methyleugenol content of ‘BaiZiZhi’ and ‘Guo’ was highest in the full-blooming stage (BZ5 and GM5), and that of ‘Hanxiang’ was highest in the withering stage (HZ7). Eugenol is catalyzed by (iso)eugenol *O*-methyltransferase to synthesize methyleugenol. Changes in the relative contents of eugenol and methyleugenol in *R. rugosa* cultivars at different flowering stages were examined. Synthesis in ‘BaiZiZhi’ started in the early-blooming stage (BZ3) and continued to the full-blooming stage (BZ5). In ‘Hanxiang’ synthesis started at the full-blooming stage (HX5) and peaked in the withering stage (HX7). In the process of synthesizing methyleugenol, (iso)eugenol *O*-methyltransferase plays a catalytic role in ‘Guo’; the relative content of methyleugenol decreased sharply in the withering stage (GM7), indicating that synthesis started in the early-blooming stage (GM3) and ended after the full-blooming stage (GM5).

## 4 Discussion

### 4.1 Industry application evaluation of *R. rugosa*

In recent years, the AHP method has been widely used in the evaluation of plant germplasm resources and application evaluation of garden plants (Zhao and Wang, 2016) and horticultural plants (Sheng et al., 2021). Selection of appropriate cultivars can ensure superior flower quality and shape. Flower type, color, and leaf shape of plants are not only important characteristics of plants that are both ornamental and edible, but also represent the most inherent phenotypic differences among cultivars. Many previous studies on the morphological traits of *R. rugosa* have been conducted (Rusanov et al., 2013). In those studies, there is little mention of the characteristics of thorns. In the present study, we paid attention to relevant elements of the flowers, fragrance characteristics, and biological characteristics of the thorns in *R. rugosa*. A comprehensive evaluation system was constructed using the AHP.

### 4.2 Comparative analysis of *R. rugosa*, fragrant *R. hybrida* cultivars, and aromatic *Rosa* species

*Rosa rugosa* was shown to be especially favorable with regard to the contents of important fragrant substances, such as rose oxide, citronellol, citronellyl acetate, and citronellal, which is consistent with previous studies (Ren et al., 2018). With regard to human health, previous studies showed that phenethyl alcohol and linalool have neuroprotective (Orhan et al., 2009; Souto-Maior et al., 2017), antioxidant (Hancianu et al., 2013; Senol et al., 2013), and anxiety-relieving effects (Umezu et al., 2002; Linck et al., 2010), and citronellol has anti-inflammatory (Su et al., 2010) and therapeutic effects on allergic diseases (Kobayashi et al., 2016; Pina et al., 2019). Compared with *R. rugosa*, fragrant *R. hybrida* cultivars contain lower contents or lack important aromatic substances, which is an important reason why the fragrance of *R. hybrida* is generally not as strong as that of *R. rugosa*. Compared with other scented *Rosa* species, *R. rugosa* offers no obvious advantage in phenotypic characters, but the contents of important fragrant substances are significantly higher than those of other species. Consideration should be given to crossing *R. rugosa* with other species of *Rosa* with desirable aesthetic traits and strong fragrance. *Rosa* × *damascena* is the hybrid between *R. gallica* and *R. phoenicia*. Modern investigations of *R.* × *damascena* have confirmed its antiviral, antibacterial, anticancer, antidepressant, antioxidant (Ayati et al., 2018), analgesic, anti-inflammatory, and anticonvulsant activities, and its relaxant and hypnotic effects (Mahboubi, 2016). In recent years, greater attention has focused on the essential oil and pharmacology of *R. damascena* (Dadkhah et al., 2019; Akram et al., 2020; Koohpayeh et al., 2021). *Rosa* × *damascena*, *R. hybrida* ‘Aunt Margy’s’, and *R. hybrida* ‘Dream of Garden’ could be used as alternative cultivars for cross-breeding with *R. rugosa* to optimize other phenotypic traits of *R. rugosa*. *Rosa rugosa* ‘Guo’ is a wild selection of *R. rugosa* with outstanding fragrance characteristics and could be crossed with other scented *Rosa* species with favorable traits, such as *R.* × *damascena* ‘Alba’, *R. gallica*, and *R.* ‘Dianhong’, to improve its qualities. The crossing of different germplasm to obtain an ideal hybrid combination has always been the focus of breeding. The more distant the genetic relationship of the parents, the more marked the heterosis. Cluster analysis can clarify the genetic relationship between germplasm, and guide the combination and prediction of dominant hybrids. *Rosa* germplasm represents the multidirectionality of diversity and genetic divergence, which is conducive to efficient hybrid breeding (Sen et al., 2021).

### 4.3 Target metabolites and metabolites

The main floral components, such as citronellol, phenethyl alcohol, nerol, and linalool, have a major impact on plant quality. The difference between *R. rugosa* and other species of *Rosa* is largely the contribution of fragrance substances. We screened the predominant floral scent components, as well as differential metabolites and the metabolic pathways involved. Metabolite information was acquired at four stages of floral development (flower-bud stage, early-blooming stage, full-blooming stage, and withering stage) in the *R. rugosa* cultivars ‘BaiZiZhi’, ‘Hanxiang’, and ‘Guo’. The results showed that the highest content of all fragrance components was detected at the full-blooming stage, which was consistent with previous studies (Sun et al., 2019). The relative contents of fragrance components in ‘Hanxiang’ were generally higher than those of the other two *R. rugosa* cultivars, and the contents of terpenoids and a small number of aldehydes and heterocyclic substances were higher at the flower-bud stage than in the early-blooming stage. This has not been reported previously. It is speculated that it may be caused by genetic differences in ‘Hanxiang’, which needs further research. By studying biological characteristics and fragrance components of different *R. rugosa* cultivars, genotypes more suitable for edible and medicinal application may be selected.

The synthesis of aromatic amino acids is unique to the metabolism of plants and microorganisms. A detailed understanding of their synthesis and metabolism may promote the consolidation and expansion of knowledge of the metabolism of living organisms. Most changes in floral fragrance components are caused by catalysis by relevant enzymes. Citronellol, nerol, geraniol, linalool, phenethyl alcohol, and eugenol are especially important aromatic substances in *R. rugosa*. The terpene precursor isopentenyl diphosphate is synthesized in the mevalonate (MVA) pathway in the cytoplasm, and the 1-deoxy-D-xylulose-5-phosphate (DXP) pathway or methylerythritol 4-phosphate (MEP) pathway in plastids (Wei et al., 2017). Further study on the contribution of these two pathways to terpene biosynthesis showed that *R. rugosa* uses the cytoplasmic MVA pathway to synthesize monoterpene precursors. In addition, although many genes in the MVA and MEP pathways are well known in diverse plant species, limited information is available for *R. rugosa*, which requires further exploration. Geranyl diphosphate (GPP) is the precursor of various monoterpene compounds under the action of different monoterpene synthases (Lichtenthaler, 1999). By comparing VOC characteristics and differential gene expression of fragrant and non-fragrant rose cultivars, Sun et al. (2016) observed that geraniol synthesis involved a Nudix hydrolase (RhNUDX1). Phosphoenolpyruvate and erythrose 4-phosphate are common substrates for the synthesis of aromatic amino acids, which thus involves the phenylalanine, tyrosine, and tryptophan synthesis pathway. There are two synthetic pathways for 2-phenethyl alcohol in *R. rugosa.* The first involves phenylacetaldehyde synthase and phenylacetaldehyde reductase (PAR); the second involves aromatic amino acid aminotransferase, phenylpyruvate decarboxylase, and PAR. The former synthesis pathway was detected in the present study.

We described changes in differential metabolites in different cultivars of *R. rugosa* and at different stages of floral development. Enzymes play an important role in the two enriched metabolic pathways. These findings provide a foundation for future breeding of *R. rugosa* and the mining of fragrance-related candidate genes

## Data availability statement

The raw data supporting the conclusions of this article will be made available by the authors, without undue reservation.

## Author contributions

XC: Conceptualization, Methodology, Formal analysis, Resources, Writing-Reviewing and Editing, Funding acquisition. YF: Investigation, Data Curation, Writing-Original draft preparation. DC: Writing-Reviewing, Validation. CL: Writing- Reviewing. XY: Methodology, Writing-Reviewing, Supervision. CH: Supervision, Funding acquisition, Project administration. All authors contributed to the article and approved the submitted version.

## Funding

This work was supported by funds from the Beijing Academy of Agriculture and Forestry Sciences (KJCX20200801, KJCX20220103) and the Beijing Innovation Consortium of Agriculture Research System (BAIC09-2022).

## Acknowledgments

The authors thank MetWare for conducting targeted metabolome and volatile metabolome analyses for this study. The authors also thank Robert McKenzie, PhD, from Liwen Bianji (Edanz) (www.liwenbianji.cn) for editing the English text of a draft of this manuscript.

## Conflict of interest

The authors declare that the research was conducted in the absence of any commercial or financial relationships that could be construed as a potential conflict of interest.

## Publisher’s note

All claims expressed in this article are solely those of the authors and do not necessarily represent those of their affiliated organizations, or those of the publisher, the editors and the reviewers. Any product that may be evaluated in this article, or claim that may be made by its manufacturer, is not guaranteed or endorsed by the publisher.

## Supplementary material

Supplementary Figure 1 Detected integral graph of citronellol.

Supplementary Figure 2 Detected integral graph of phenethyl alcohol.

Supplementary Figure 3 Detected integral graph of farnesol.

Supplementary Figure 4 Detected integral graph of nerol.

Supplementary Figure 5 Detected integral graph of rose oxide.

Supplementary Figure 6 Heatmap of 27 *R. rugosa*, 43 scented *R. hybrida*, and seven aromatic *R.* species. Morphological characters (*x*-axis) are represented for the 77 *Rosa* accessions (*y*-axis).

Supplementary Figure 7 Superimposed graph of the total ions current diagram of the quality-control mixed sample (QC Mix). Total ions current (TIC) was measured with the QC Mix sample. The *x*-axis is retention time (Rt) of the volatile substances in the sample during detection, and the *y*-axis is the ion current intensity of ion detection (the unit of intensity is counts per second [cps]).

Supplementary Figure 8 Principal component analysis scatterplot of the first and second principal components (PC1 and PC2, respectively). Each circle represents an individual sample; different samples are indicated by different colors.

Supplementary Figure 9 S-plot of OPLS-DA between groups. The *x*-axis is the covariance between the principal component and the metabolite, and the *y*-axis is the correlation coefficient between the principal component and the metabolite. Red dots indicate VIP ≥ 1 for these metabolites, and green dots indicate VIP < 1 for these metabolites.

Supplementary Figure 10 Volcano map of differential metabolites. Each point in the volcano plot represents a metabolite, the *x*-axis is the logarithm of the quantitative fold difference of a metabolite in two samples, and the *y*-axis is the VIP value. The larger the absolute value on the *x*-axis, the greater the fold difference of expression between two samples. The larger the *y*-axis value, the more strongly significant the differential expression, and the more reliable the differentially expressed metabolites obtained by screening. Green dots represent down-regulated differentially expressed metabolites, red dots represent up-regulated differentially expressed metabolites, and gray dots represent metabolites detected but not significantly different.

Supplementary Figure 11 Differential metabolite KEGG pathway enrichment map. The *x*-axis is the Rich factor corresponding to each pathway, and the *y*-axis is the pathway name. The color of the point indicates the *P*-value – the more intensely red, the more significant the enrichment. The size of dots represents the number of enriched differential metabolites. The Rich factor is the ratio of the number of differentially expressed metabolites in the corresponding pathway to the total number of metabolites detected and annotated by the pathway. A higher value indicates a greater degree of enrichment. Pvalue is the *p-*value from the hypergeometric test.

## References

[B1] Akram M. Riaz M. Munir N. Akhter N. Zafar S. Jabeen F.. (2020). Chemical constituents, experimental and clinical pharmacology of *Rosa damascena*: A literature review. J. Pharm. Pharmacol. 72 (2), 161–174. doi: 10.1111/jphp.13185 31709541

[B2] Alizadeh Z. Fattahi M. (2021). Essential oil, total phenolic, flavonoids, anthocyanins, carotenoids and antioxidant activity of cultivated damask rose (*Rosa damascena*) from Iran: With chemotyping approach concerning morphology and composition. Sci. Hortic. 288, 110341. doi: 10.1016/j.scienta.2021.110341

[B3] Ayati Z. Amiri M. S. Ramezani M. Delshad E. Sahebkar A. SahebkarEmami A.. (2018). Phytochemistry, traditional uses and pharmacological profile of rose hip: A review. Curr. Pharm. Des. 24 (35), 4101–4124. doi: 10.2174/1381612824666181010151849 30317989

[B4] Chen C. J. Chen H. Zhang Y. Thomas H. R. Frank M. H. He Y. H.. (2020). TBtools: An integrative toolkit developed for interactive analyses of big biological data. Mol. Plant 13 (8), 1194–1202. doi: 10.1016/j.molp.2020.06.009 32585190

[B5] Dadkhah A. Fatemi F. Malayeri M. R.M. Ashtiyani M. H.K. Noureini S. K. Rasooli A. (2019). Considering the effect of *Rosa damascena* mill. essential oil on oxidative stress and COX-2 gene expression in the liver of septic rats. Turk. J. Pharm. Sci. 16 (4), 416. doi: 10.4274/tjps.galenos.2018.58815 32454744 PMC7227890

[B6] Dani K. G. S. Fineschi S. Michelozzi M. Trivellini A. Pollastri S. Loreto F. (2021). Diversification of petal monoterpene profiles during floral development and senescence in wild roses: Relationships among geraniol content, petal colour, and floral lifespan. Oecologia 197 (4), 957–969. doi: 10.1007/s00442-020-04710-z 32712874 PMC8591013

[B7] Farre-Armengol G. Filella I. Llusia J Penuelas J. (2013). Floral volatile organic compounds: Between attraction and deterrence of visitors under global change. Perspect. Plant Ecol. 15 (1), 56–67. doi: 10.1016/j.ppees.2012.12.002

[B8] Feng L. G. Chen C. Li T. L. Wang M. Tao J. Zhao D. Q.. (2014). Flowery odor formation revealed by differential expression of monoterpene biosynthetic genes and monoterpene accumulation in rose (*Rosa rugosa* thunb.). Plant Physiol. Biochem. 75, 80–88. doi: 10.1016/j.plaphy.2013.12.006 24384414

[B9] Hancianu M. Cioanca O. Mihasan M. Hritcu L. (2013). Neuroprotective effects of inhaled lavender oil on scopolamine-induced dementia *via* anti-oxidative activities in rats. Phytomedicine 20 (5), 446–452. doi: 10.1016/j.phymed.2012.12.005 23351960

[B10] Kobayashi Y. Sato H. Yorita M. Nakayama H. Miyazato H. Sugimoto K.. (2016). Inhibitory effects of geranium essential oil and its major component, citronellol, on degranulation and cytokine production by mast cells. Biosci. Biotechnol. Biochem. 80 (6), 1172–1178. doi: 10.1080/09168451.2016.1148573 26927807

[B11] Koohpayeh S. A. Hosseini M. Nasiri M. Rezaei M. (2021). Effects of *Rosa damascena* (Damask rose) on menstruation-related pain, headache, fatigue, anxiety, and bloating: A systematic review and meta-analysis of randomized controlled trials. J. Health Promotion. Iinc. 10:272. doi: 10.4103/jehp.jehp_18_21 PMC8395985 34485569

[B12] Lichtenthaler H. K. (1999). The 1-deoxy-D-xylulose-5-phosphate pathway of isoprenoid biosynthesis in plants. Annu. Rev. Plant Biol. 50 (1), 47–65. doi: 10.1146/annurev.arplant.50.1.47 15012203

[B13] Linck V. M. Silvaad A. L. Figueiróab M. Caramãoe E. B. Morenof P. R. H. Elisabetskyabc E. (2010). Effects of inhaled linaloolin anxiety, social interaction and aggressive behavior in mice. Phytomedicine 17 (8), 679. doi: 10.1016/j.phymed.2009.10.002 19962290

[B14] Magnard J. L. Roccia A. Caissard J. C. Vergne P. Sun P. L. Hecquet R.. (2015). Biosynthesis of monoterpene scent compounds in roses. Science 349 (6243), 81–83. doi: 10.1126/science.aab0696 26138978

[B15] Mahboubi M. (2016). *Rosa damascena* as holy ancient herb with novel applications. J. Tradit. Compl. Med. 6 (1), 10–16. doi: 10.1016/j.jtcme.2015.09.005 PMC4737971 26870673

[B16] Mostafavi A. S. Omidi M. Azizinezhad R. Etminan A. Badi H. N. (2021). Genetic diversity analysis in a mini core collection of damask rose (*Rosa damascena* mill.) germplasm from Iran using URP and SCoT markers. J. Genet. Eng. Biotechnol. 19 (1), 1–14. doi: 10.1186/s43141-021-00247-7 34591207 PMC8484433

[B17] Orhan G. Orhan I. Subutay-Oztekin N. Fikri A. K. Sener B. (2009). Contemporary anticholinesterase pharmaceuticals of natural origin and their synthetic analogues for the treatment of alzheimers disease. Recent Pat. CNS Drug Discovery 4 (1), 43–51. doi: 10.2174/157488909787002582 19149713

[B18] Pina L. T. S. Ferro J. N.S. Rabelo T. K. Oliveira M. A. Scotti L. Scotti M. T.. (2019). Alcoholic monoterpenes found in essential oil of aromatic spices reduce allergic inflammation by the modulation of inflammatory cytokines. Nat. Prod. Res. 33 (12), 1773–1777. doi: 10.1080/14786419.2018.1434634 29394874

[B19] Raymond O. Gouzy J. Just J. Badouin H. Verdenaud M. Lemainque A.. (2018). The *Rosa* genome provides new insights into the domestication of modern roses. Nat. Genet. 50 (6), 772–777. doi: 10.1038/s41588-018-0110-3 29713014 PMC5984618

[B20] Ren G. Xue P. Sun X. Y. Zhao G. (2018). Determination of the volatile and polyphenol constituents and the antimicrobial, antioxidant, and tyrosinase inhibitory activities of the bioactive compounds from the by-product of *Rosa rugosa* thunb. var. plena regal tea. BMC Complementary. Altern. Med. 18 (1), 1–9. doi: 10.1186/s12906-018-2374-7 PMC6247689 30458808

[B21] Rusanov K. Kovacheva N. Rusanova M. Atanassov I. (2013). Flower phenotype variation, essential oil variation and genetic diversity among *Rosa* alba l. accessions used for rose oil production in Bulgaria. Sci. Hortic. 161, 76–80. doi: 10.1016/j.scienta.2013.07.010

[B22] Sen N. Biswas K. Sinha S. N. (2021). Assessment of genetic divergence through cluster analysis of chilli varieties. J. Biosci. 46 (3), 1–6. doi: 10.1007/s12038-021-00167-1 34148875

[B23] Senol F. S. Orhan I. E. Kurkcuoglu M. Khan M. T.H. Altintas A. Sener B.. (2013). A mechanistic investigation on anticholinesterase and antioxidant effects of rose (*Rosa damascena* mill.). Food Res. Int. 53 (1), 502–509. doi: 10.1016/j.foodres.2013.05.031

[B24] Sheng L. Ni Y. A. Wang J. W. Chen Y . Gao H. S.. (2021). Characteristic-aroma-component-based evaluation and classification of strawberry varieties by aroma type. Molecules 26 (20) 6219. doi: 10.3390/molecules26206219 34684796 PMC8540309

[B25] Souto-Maior F. N. da Fonseca D. V. Salgado P. R. R. Monte L. D. de Sousa D. P. de Almeida R. N. (2017). Antinociceptive and anticonvulsant effects of the monoterpene linalool oxide. Pharm. Biol. 55 (1), 63–67. doi: 10.1080/13880209.2016.1228682 27622736 PMC7012048

[B26] Su Y. W. Chao S. H. Lee M. H. Ou T. Y. Tsai Y. C. Su Y. W.. (2010). Inhibitory effects of citronellol and geraniol on nitric oxide and prostaglandin e-2 production in macrophages. Planta. Med. 76 (15), 1666–1671. doi: 10.1055/s-0030-1249947 20506077

[B27] Sun P. Schuurink R. C. Caissard J. C. Hugueney P. Baudino S. (2016). My way: noncanonical biosynthesis pathways for plant volatiles. Trends Plant Sci. 21 (10), 884–894. doi: 10.1016/j.tplants.2016.07.007 27475252

[B28] Sun Y. R. Wang W. L. Zhao L. Y. Zheng C. S. Ma F. F. (2019). Changes in volatile organic compounds and differential expression of aroma-related genes during flowering of Rosa rugosa ‘Shanxian’. Hortic. Environ. Biotechnol. 60 (5), 741–751. doi: 10.1007/s13580-019-00166-0

[B29] Umezu T. Ito H. Nagano K. Yamakoshi M. Oouchi H. Sakaniwa M.. (2002). Anticonflict effects of rose oil and identification of its active constituents. Life Sci. 72 (1), 91–102. doi: 10.1016/S0024-3205(02)02197-5 12409148

[B30] Wang Q. Wang D. Zhang R. M. Gao Y. (2014). Changes in constituents and contents of volatile organic compounds in wisteria floribunda at three flowering stages. J. Zhejiang. A&F. Univ. 31 (4), 647–653. doi: 10.11833/j.issn.2095-0756.2014.04.023

[B31] Wang H. Yao L. (2013). Comparative analysis of components obtained from rose petals by direct thermal desorption and water distillation. J. Shanghai. Jiaotong. Univ. (Agric. Sci.) 31 (1), 46–51. doi: 10.3969/J.ISSN.1671-9964.2013.01.009

[B32] Wei T. t. Zang S. Zeng Y. Q. Chen C. Li T. L. Feng L. G. (2017). Metabolic regulation of monoterpene compounds in plants. J. Yangzhou. Univ. (Agric. Life Sci.) 38 (2), 122–126. doi: 10.16872/j.cnki.1671-4652.2017.02.022

[B33] Xu J. J. Zang F. Q. Wu Q. C. Wang Y. Wang B. S. Huang P.. (2021). Analysis of the genetic diversity and molecular phylogeography of the endangered wild rose (*Rosa rugosa*) in China based on chloroplast genes. Glob. Ecol. Conserv. 28, e01653. doi: 10.1016/j.gecco.2021.e01653

[B34] Yang C. Y. Ma Y. J. Cheng B. X. Zhou L. J. Yu C. Luo L.. (2012). Cloning and expression analysis of eugenol synthase gene (*RhEGS1*) in cut rose (*Rosa hybrida*). Sci. Agric. Sin. 45 (3), 590–597. doi: 10.1016/j.gecco.2021.e01653

[B35] Yang C. Ma Y. J. Cheng B. X. Zhou L. J. Yu C. Luo L.. (2020). Molecular evidence for hybrid origin and phenotypic variation of *Rosa* section chinenses. Genes 11 (9), 20–26. doi: 10.3390/genes11090996 PMC7564265 32854427

[B36] Yuan N. Qiong X. Jian Z. Yude W. Lilan D. Deng F. L.. (2018). Determination of aromatic components of *Rosa davurica* pall, by headspace solid phase microextraction combined with GC-MS. Med. Plant 9 (5), 20–26. doi: 10.19600/j.cnki.issn2152-3924.2018.05.006

[B37] Yun M.-m. Li B. Y. Zhou X. M. (2016). Determination of aromatic components of flower in *Rosa rugosa* thunb. by the static headspace and gas chromatography-mass spectrometry technology. Sci. Technol. Food Ind. 37 (20), 101–103+109. doi: 10.19600/j.cnki.issn2152-3924.2018.05.006

[B38] Zhao J. L. Wang Y. W. (2016). The sustainable development research of wild plant tourism resources based on the entropy-AHP evaluation method. Adv. J. Food Sci. Technol. 10 (2), 81–89.

[B39] Zhou Y. Dai C. Y. Liu Y. Zeng J. Q. Deng L. J. Huang J. F. (2015). The analysis of aromatic compounds from *Rosa chinensis* ‘Pallida’, *R. damascene*, *R. centifolia* by GC/MS. J. Yunnan. Agric. Univ. (Nat.Sci.) 32 (01), 78–82. doi: 10.16211/j.issn.1004-390X(n).2017.01.011

[B40] Zhou L. Yu C. Cheng B. X. Han Y. Luo L. Pan H. T.. (2020a). Studies on the volatile compounds in flower extracts of *Rosa odorata* and *R. chinensis* . Ind. Crop Prod. 146, 112143. doi: 10.1016/j.indcrop.2020.112143

[B41] Zhou L. Yu C. Cheng B. X. Wan H. H. Luo L. Pan H. T.. (2020b). Volatile compound analysis and aroma evaluation of tea-scented roses in China. Ind. Crop Prod. 155 (1), 112735. doi: 10.1016/j.indcrop.2020.112735

