# Supplementary figures and images for: Evaluation of Rosa germplasm resources and analysis of floral fragrance components in R. rugosa
Source: Front Plant Sci. 2022 Oct 12;13:1026763. doi: 10.3389/fpls.2022.1026763 (PMC9597504; doi:10.3389/fpls.2022.1026763)

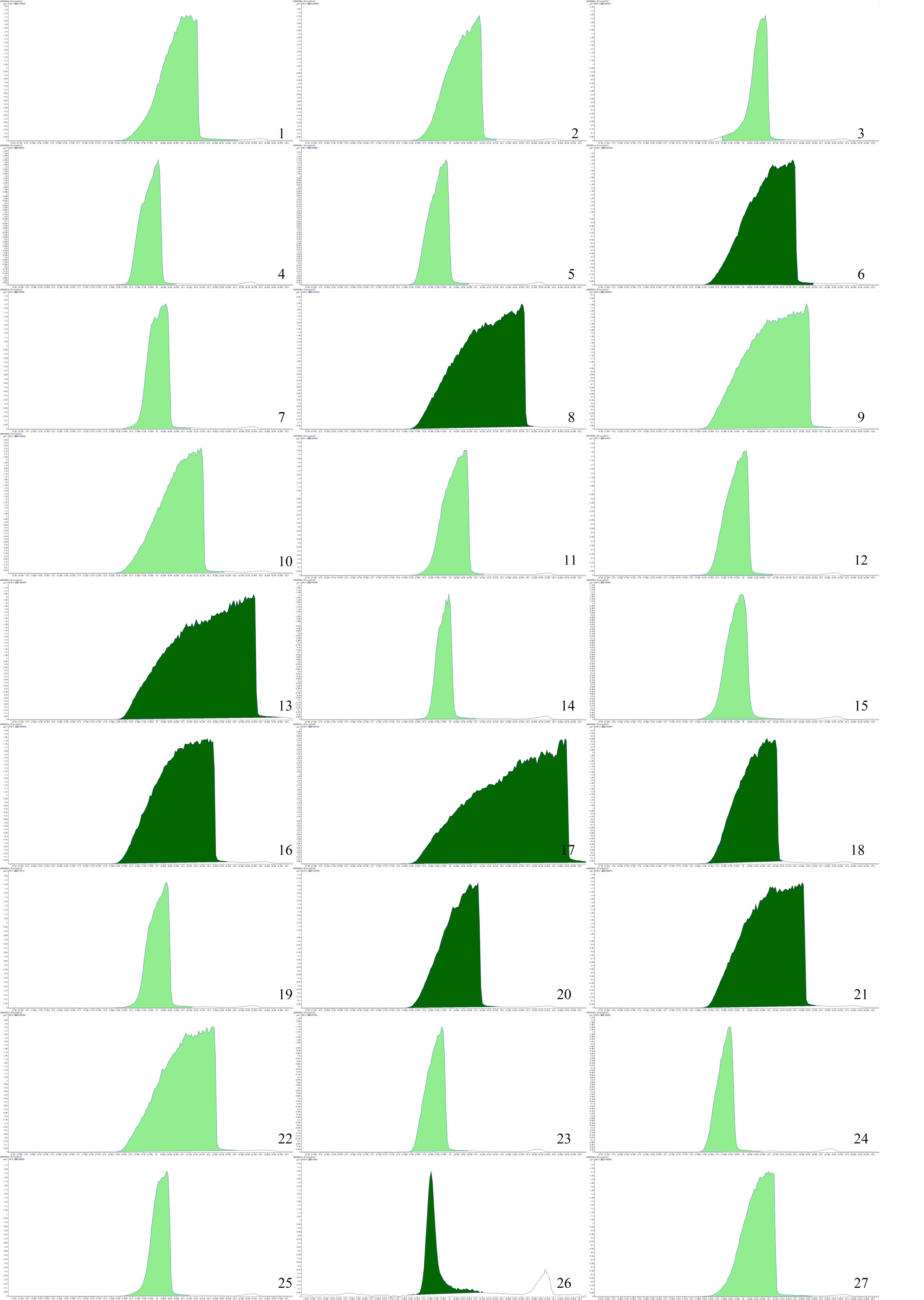

Supplement: Supplementary Figure 1 — Detected integral graph of citronellol. [file Image_1.jpeg]

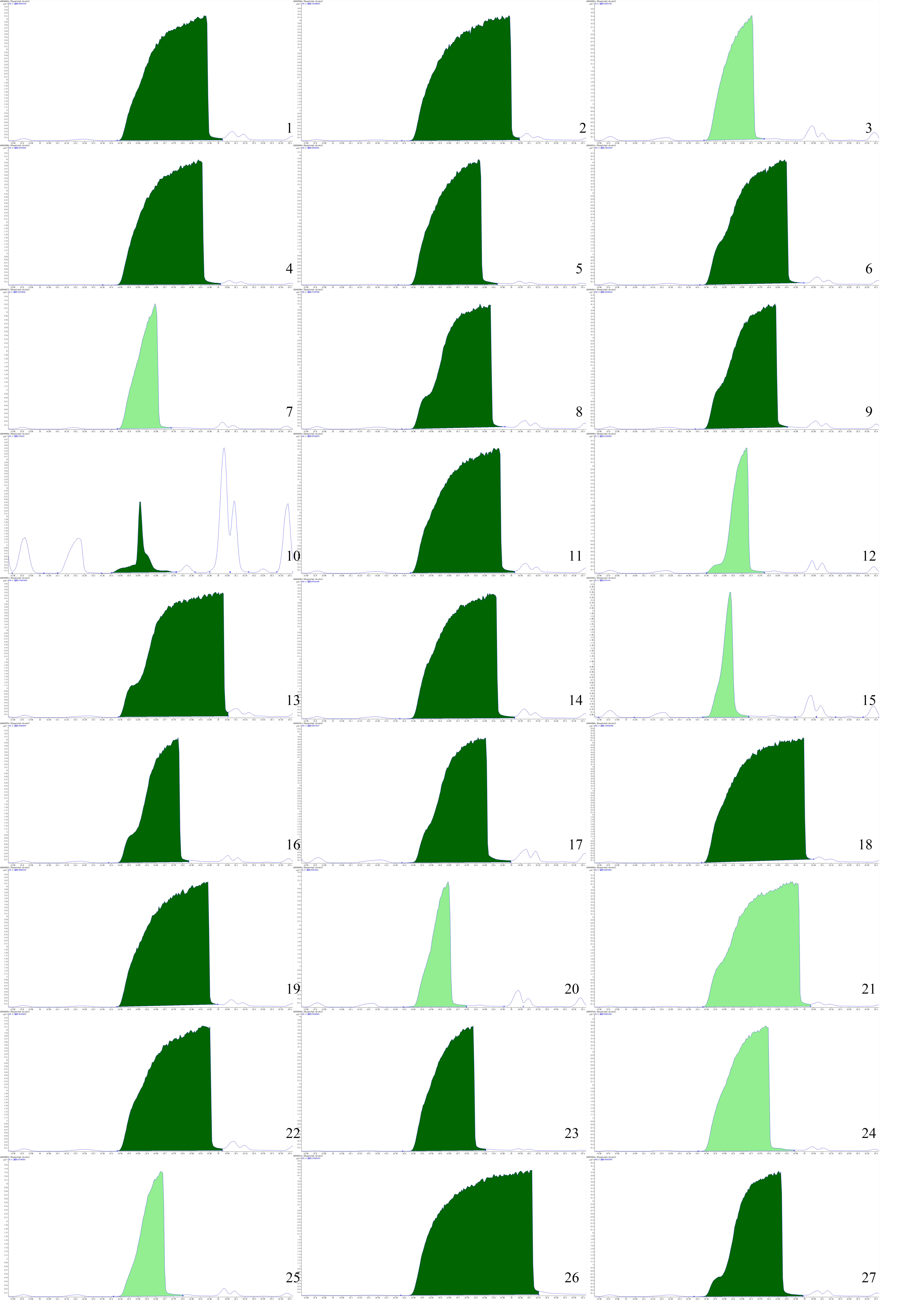

Supplement: Supplementary Figure 2 — Detected integral graph of phenethyl alcohol. [file Image_2.jpeg]

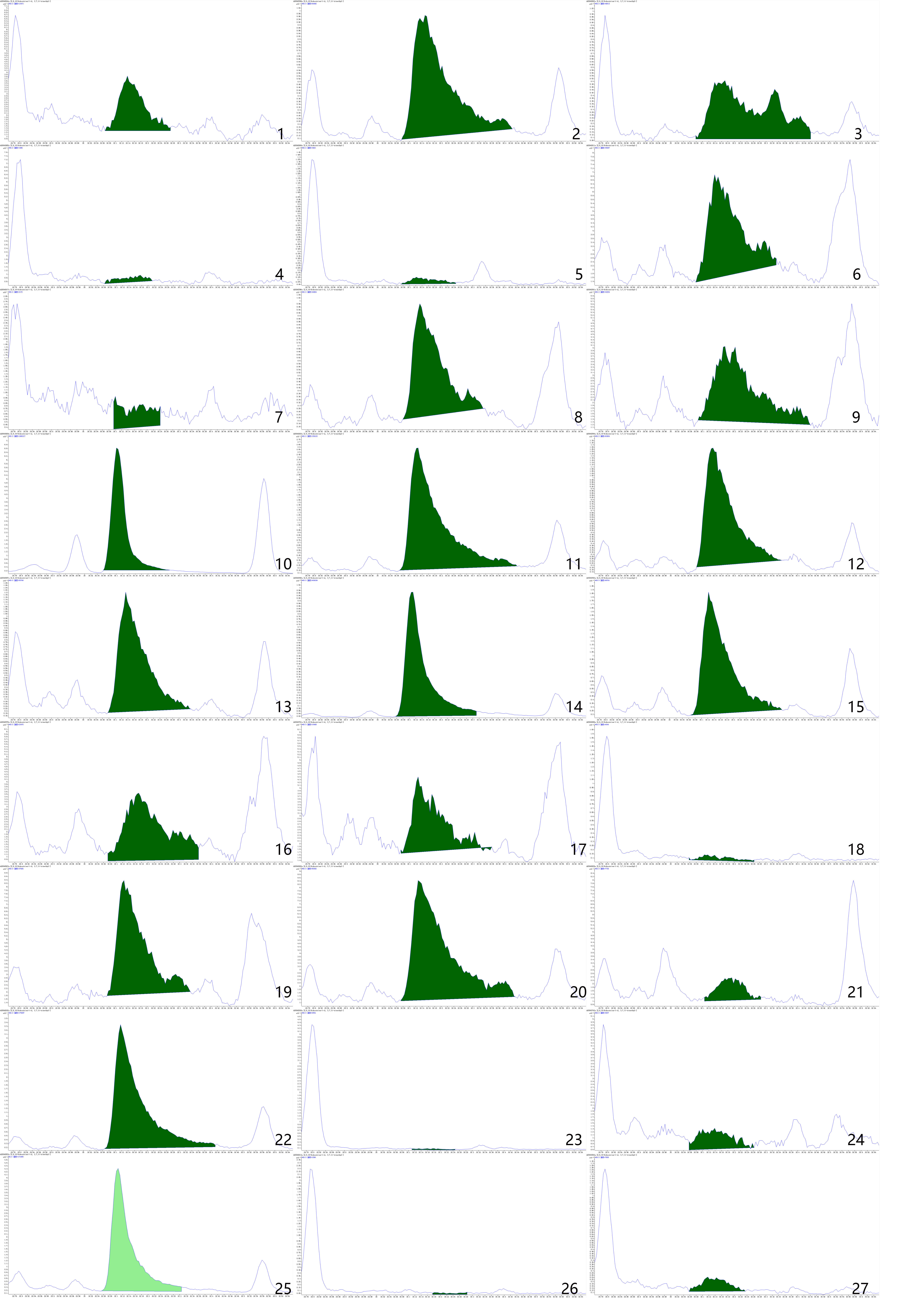

Supplement: Supplementary Figure 3 — Detected integral graph of farnesol. [file Image_3.jpeg]

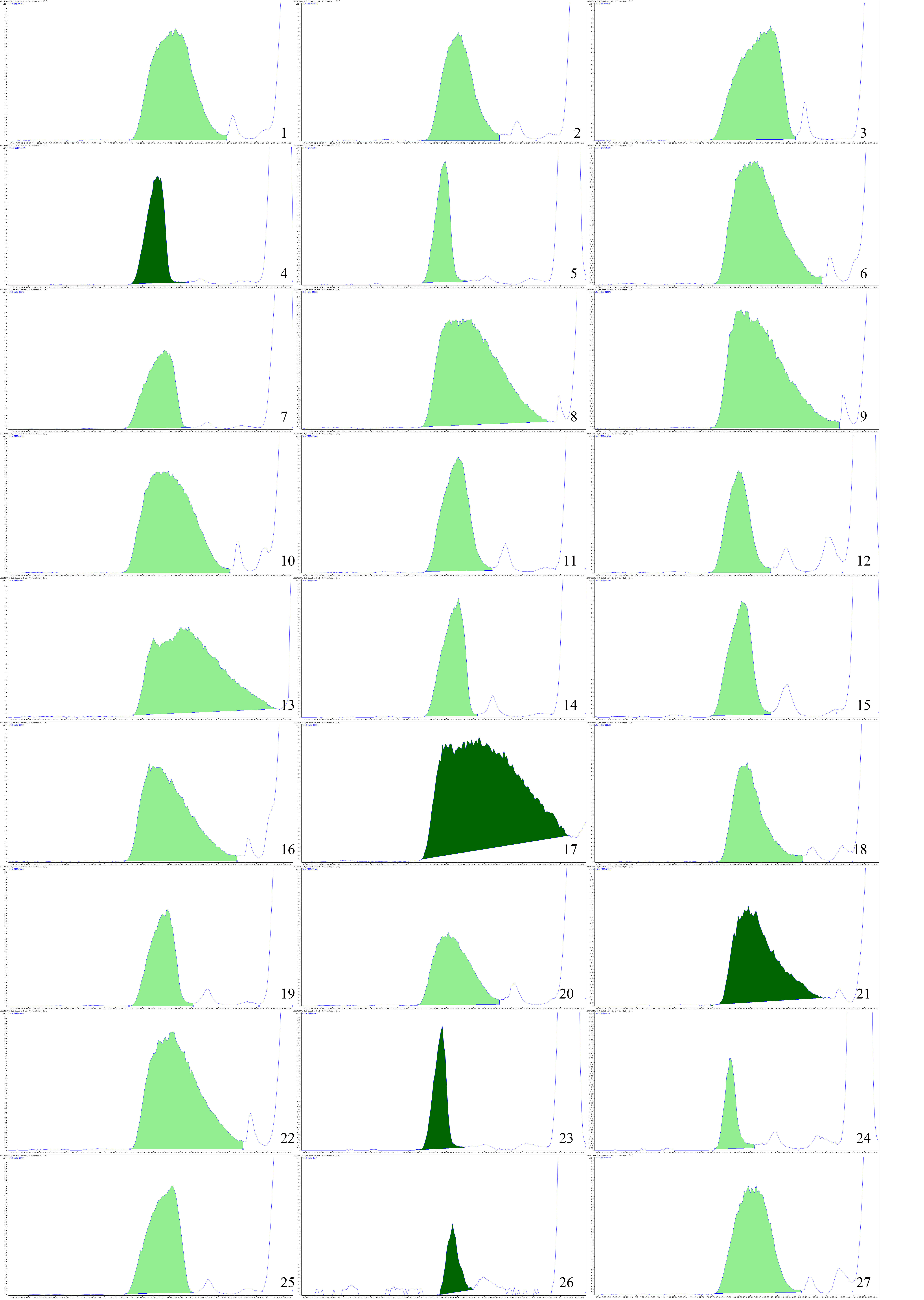

Supplement: Supplementary Figure 4 — Detected integral graph of nerol. [file Image_4.jpeg]

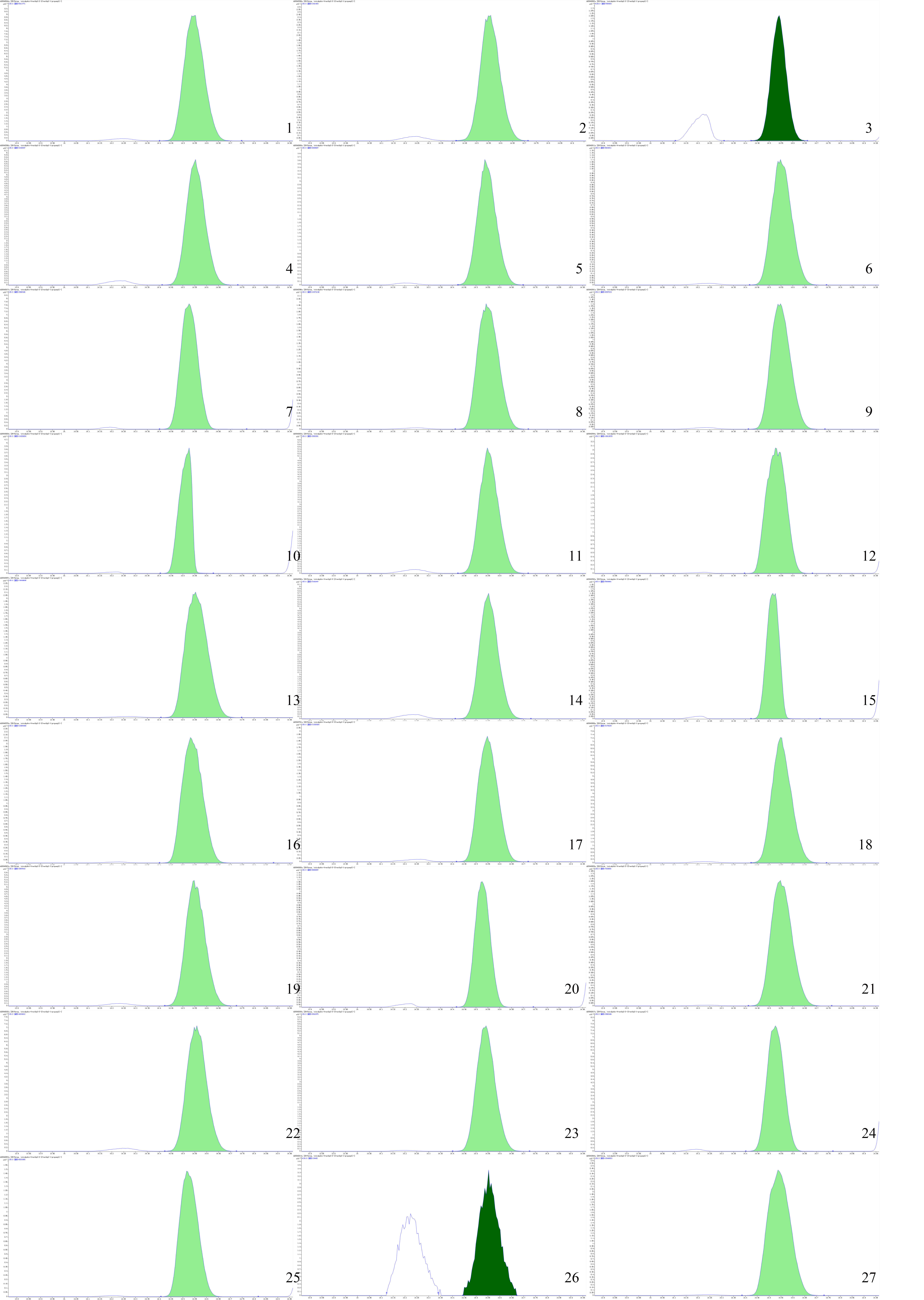

Supplement: Supplementary Figure 5 — Detected integral graph of rose oxide. [file Image_5.jpeg]

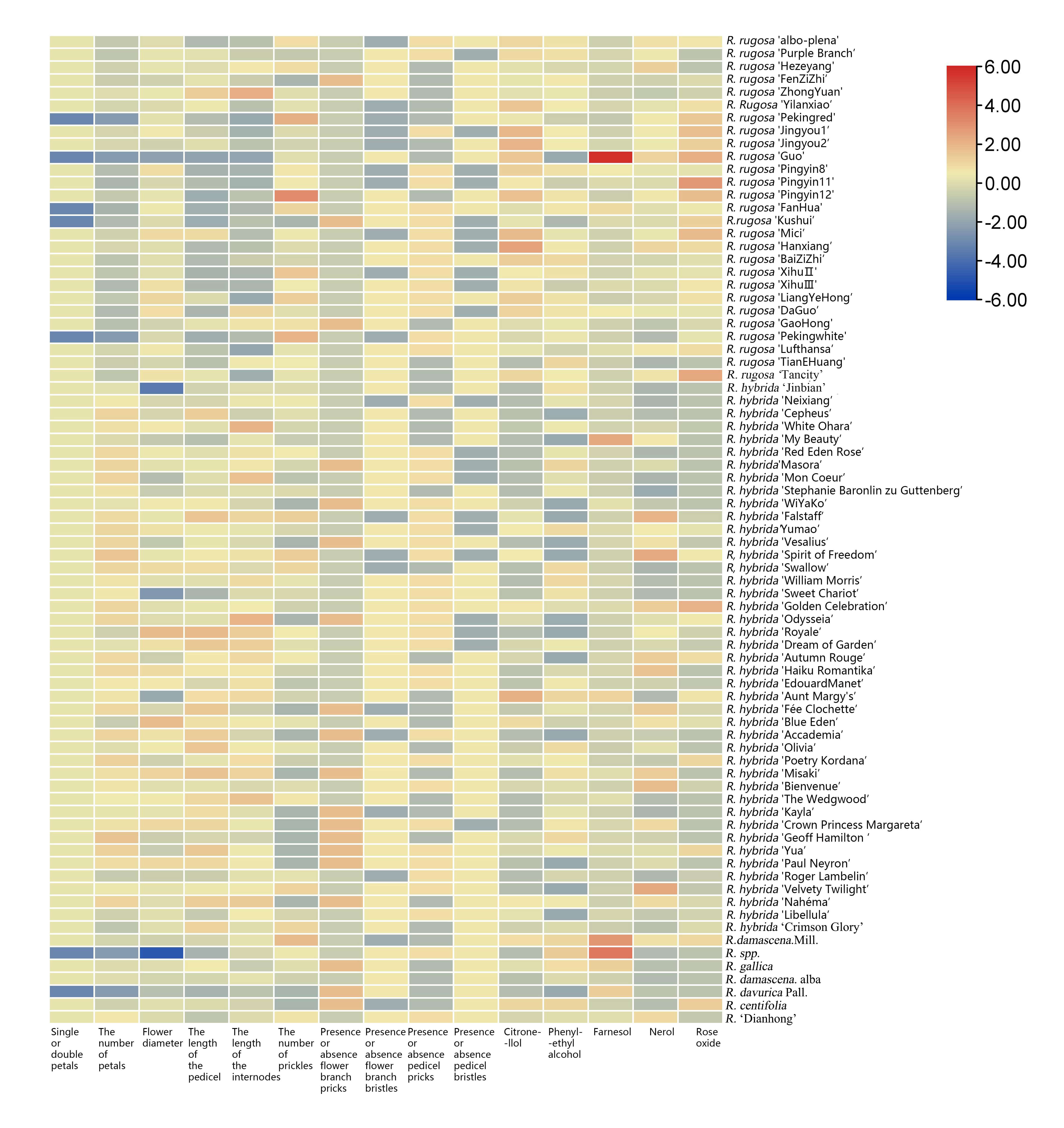

Supplement: Supplementary Figure 6 — Heatmap of 27 R. rugosa, 43 scented R. hybrida, and seven aromatic R. species. Morphological characters (x-axis) are represented for the 77 Rosa accessions (y-axis). [file Image_6.jpeg]

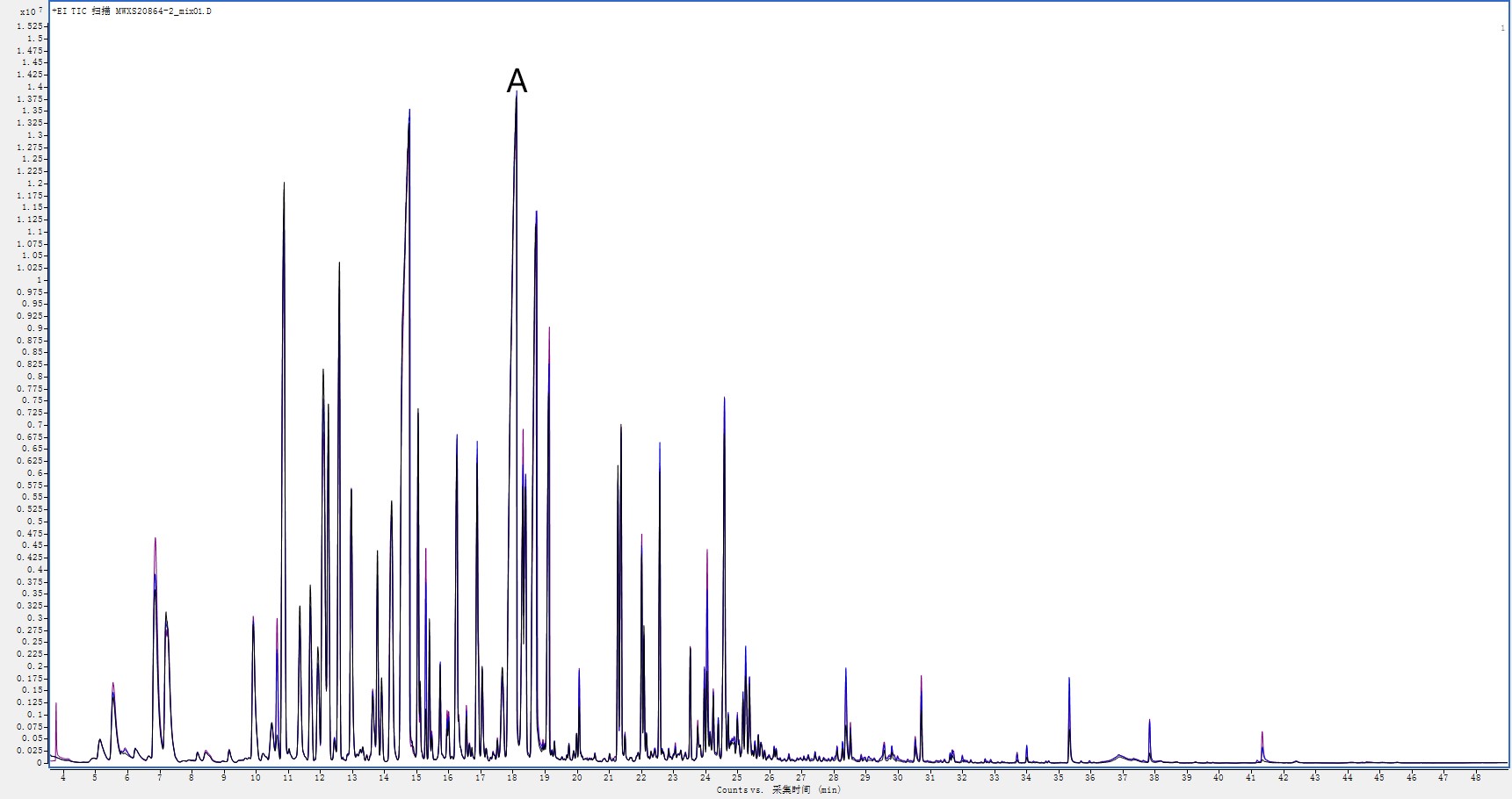

Supplement: Supplementary Figure 7 — Superimposed graph of the total ions current diagram of the quality-control mixed sample (QC Mix). Total ions current (TIC) was measured with the QC Mix sample. The x-axis is retention time (Rt) of the volatile substances in the sample during detection, and the y-axis is the ion current intensity of ion detection (the unit of intensity is counts per second [cps]). [file Image_7.jpeg]

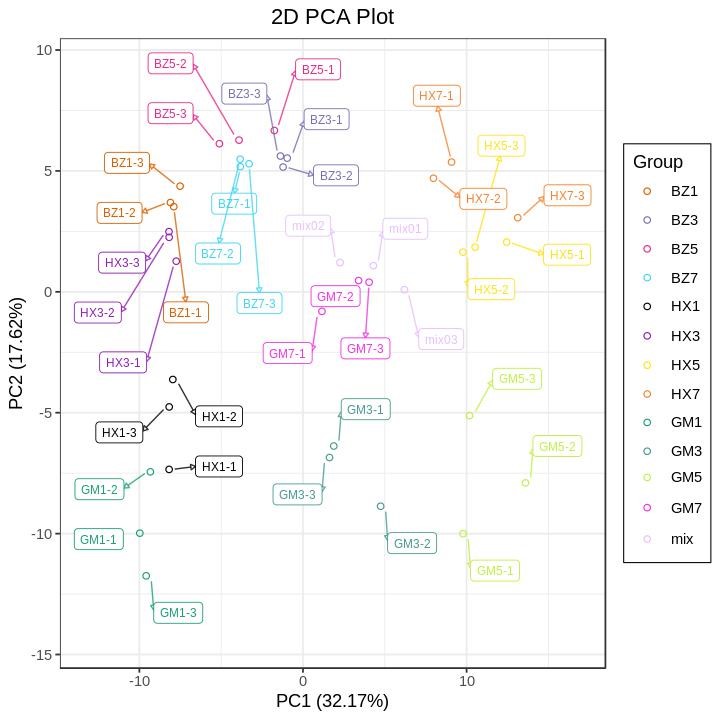

Supplement: Supplementary Figure 8 — Principal component analysis scatterplot of the first and second principal components (PC1 and PC2, respectively). Each circle represents an individual sample; different samples are indicated by different colors. [file Image_8.jpeg]

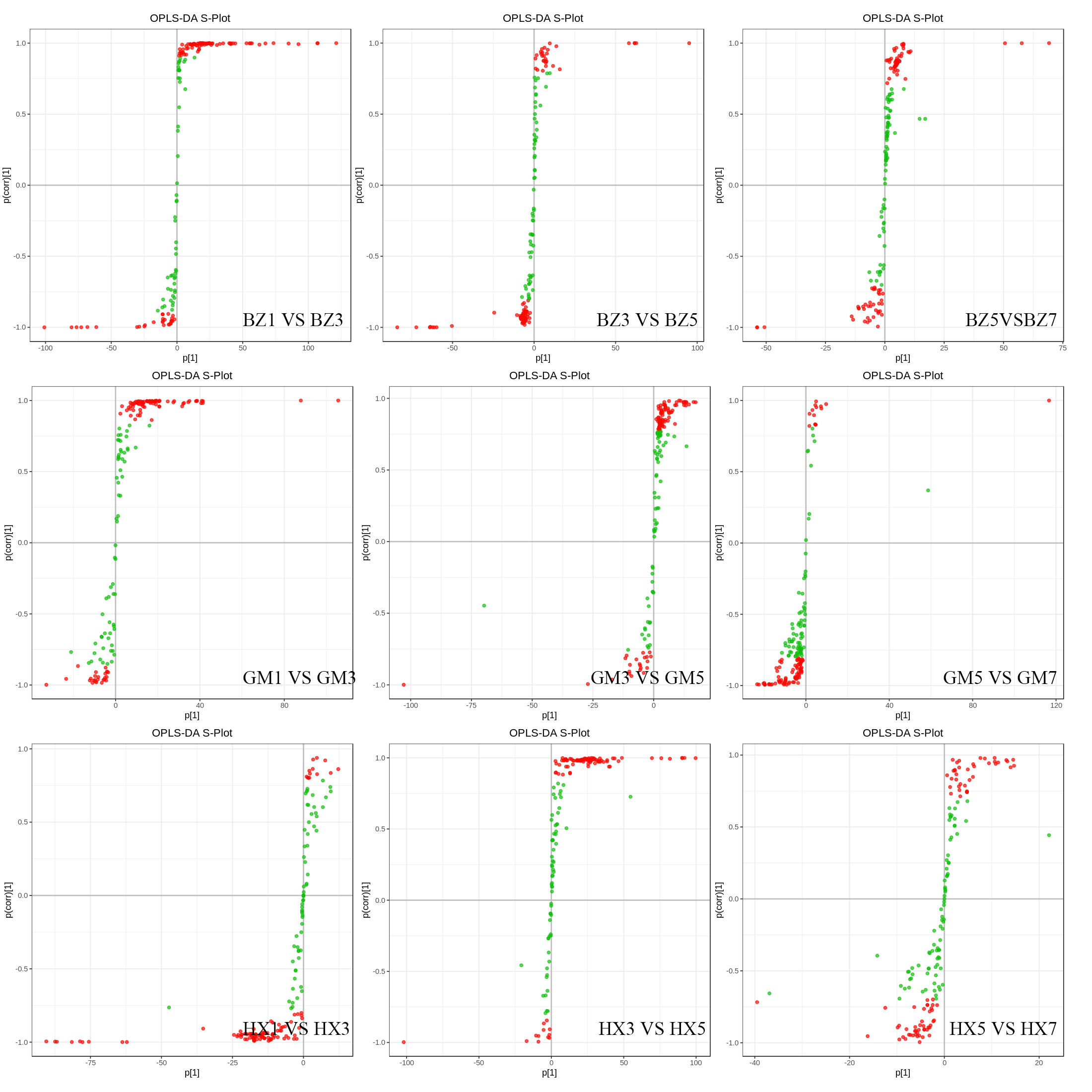

Supplement: Supplementary Figure 9 — S-plot of OPLS-DA between groups. The x-axis is the covariance between the principal component and the metabolite, and the y-axis is the correlation coefficient between the principal component and the metabolite. Red dots indicate VIP ≥ 1 for these metabolites, and green dots indicate VIP < 1 for these metabolites. [file Image_9.jpeg]

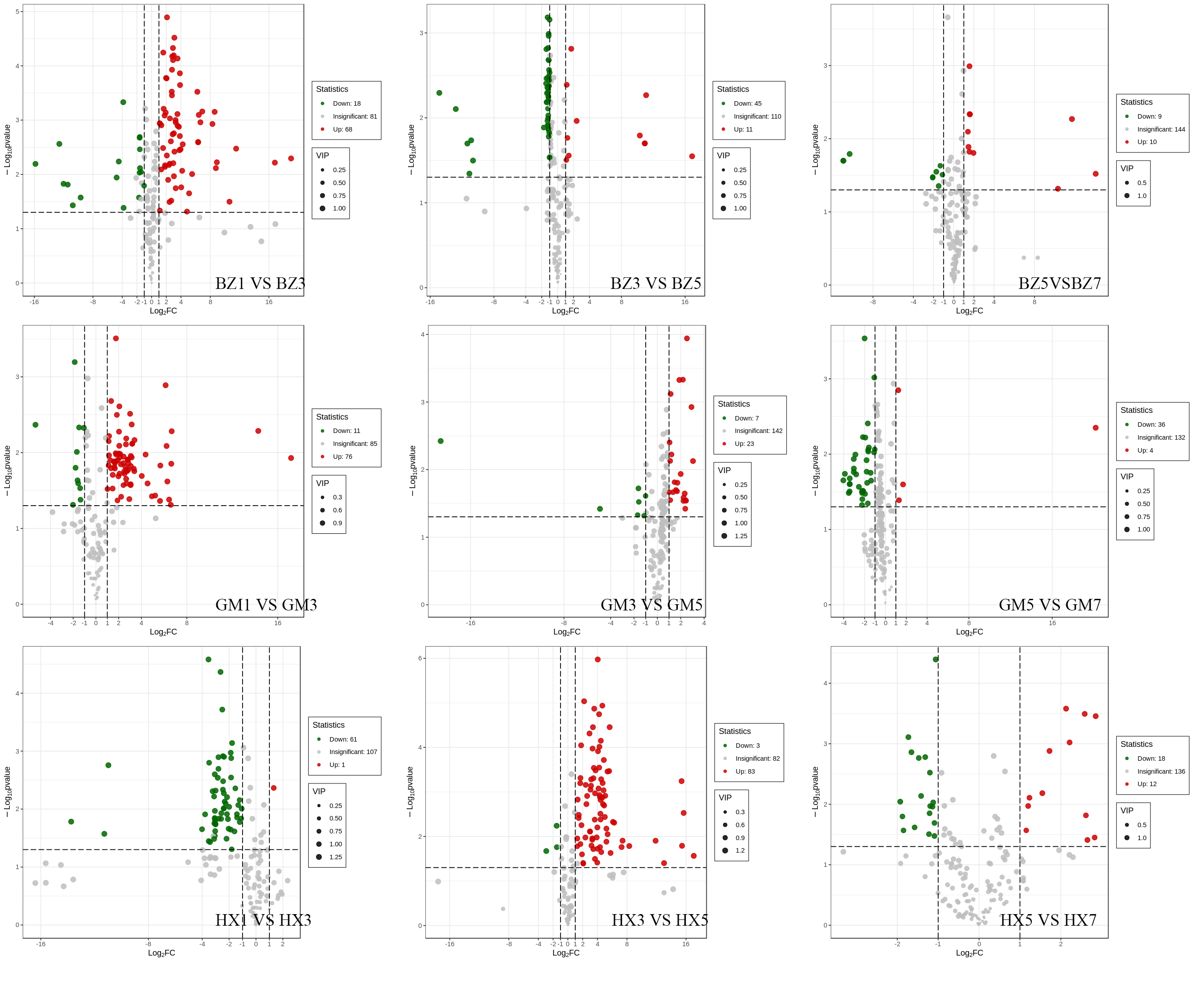

Supplement: Supplementary Figure 10 — Volcano map of differential metabolites. Each point in the volcano plot represents a metabolite, the x-axis is the logarithm of the quantitative fold difference of a metabolite in two samples, and the y-axis is the VIP value. The larger the absolute value on the x-axis, the greater the fold difference of expression between two samples. The larger the y-axis value, the more strongly significant the differential expression, and the more reliable the differentially expressed metabolites obtained by screening. Green dots represent down-regulated differentially expressed metabolites, red dots represent up-regulated differentially expressed metabolites, and gray dots represent metabolites detected but not significantly different. [file Image_10.jpeg]

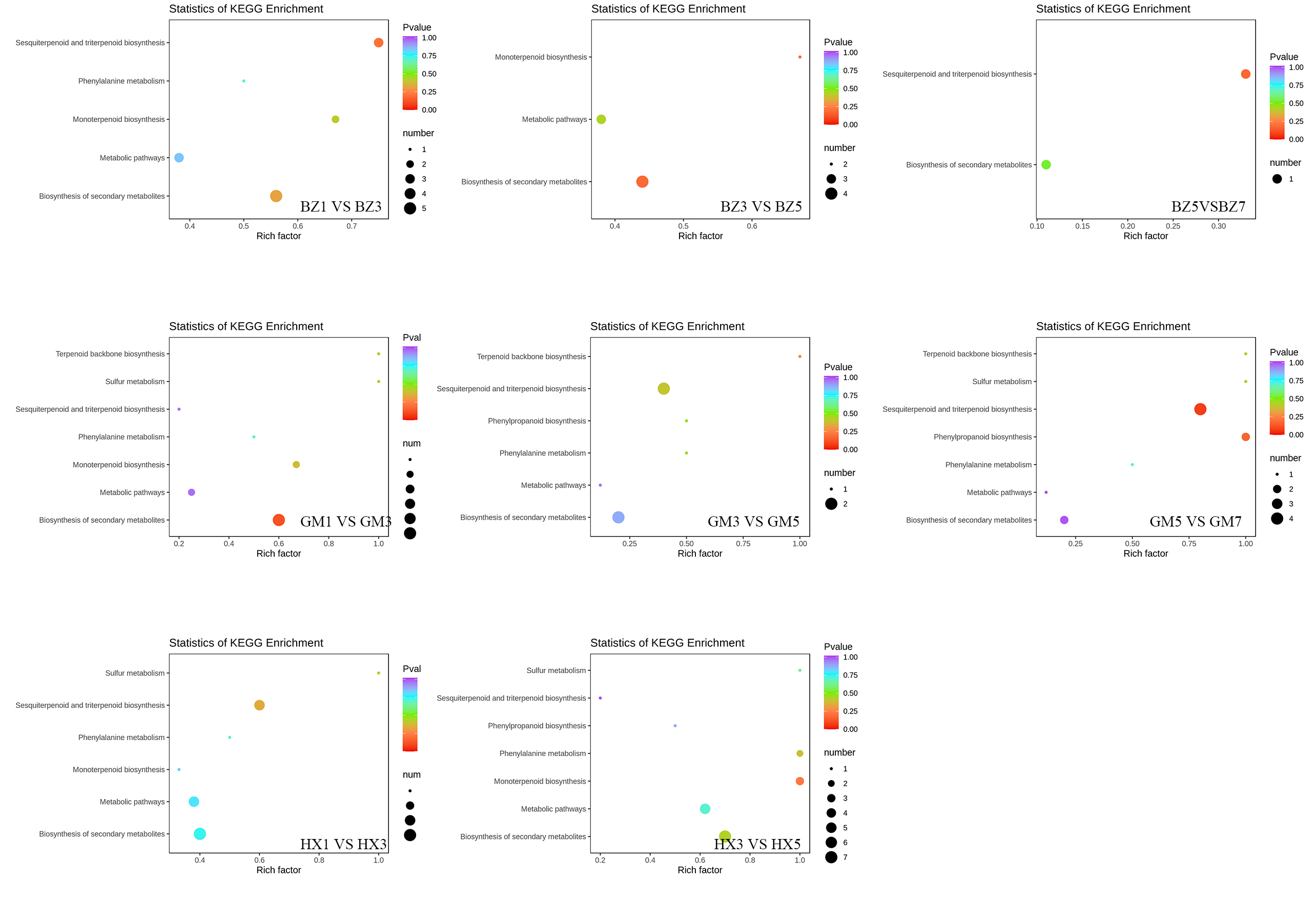

Supplement: Supplementary Figure 11 — Differential metabolite KEGG pathway enrichment map. The x-axis is the Rich factor corresponding to each pathway, and the y-axis is the pathway name. The color of the point indicates the P-value – the more intensely red, the more significant the enrichment. The size of dots represents the number of enriched differential metabolites. The Rich factor is the ratio of the number of differentially expressed metabolites in the corresponding pathway to the total number of metabolites detected and annotated by the pathway. A higher value indicates a greater degree of enrichment. Pvalue is the p-value from the hypergeometric test. [file Image_11.jpeg]
