# Supplementary material for: Evaluation of Rosa germplasm resources and analysis of floral fragrance components in R. rugosa
Source: Front Plant Sci. 2022 Oct 12;13:1026763. doi: 10.3389/fpls.2022.1026763 (PMC9597504; doi:10.3389/fpls.2022.1026763)
Supplement: Supplementary file 12 [file Table_1.docx]

**Table S1 Latin name of 27 *R. rugosa*, 43 scented *R. hybrida* cultivars and 7 classic aromatic *R.* species**

| Note | Latin name | note | Latin name | note | Latin name | note | Latin name |
| --- | --- | --- | --- | --- | --- | --- | --- |
| 1 | *R. rugosa* 'albo-plena' | 21 | *R. rugosa ‘*LiangYeHong’ | 41 | *R, hybrida* ‘Spirit of Freedom’ | 61 | *R. hybrida* ‘Kayla’ |
| 2 | *R. rugosa* ‘Purple Branch’ | 22 | *R. rugosa* ‘DaGuo’ | 42 | *R, hybrida* ‘Swallow’ | 62 | *R. hybrida* ‘Crown Princess Margareta’ |
| 3 | *R. rugosa* 'Hezeyang' | 23 | *R. rugosa* 'GaoHong' | 43 | *R. hybrida* ‘William Morris’ | 63 | *R. hybrida* ‘Geoff Hamilton ’ |
| 4 | *R. rugosa* ‘FenZiZhi’ | 24 | *R. rugosa* 'Pekingwhite' | 44 | *R. hybrida* ‘Sweet Chariot’ | 64 | *R. hybrida* ‘Yua’ |
| 5 | *R. rugosa* 'ZhongYuan' | 25 | *R. rugosa* ‘Lufthansa’ | 45 | *R. hybrida* ‘Golden Celebration’ | 65 | *R. hybrida* ‘Paul Neyron’ |
| 6 | *R. Rugosa* ‘Yilanxiao’ | 26 | *R. rugosa* 'TianEHuang' | 46 | *R. hybrida* ‘Odysseia’ | 66 | *R. hybrida* ‘Roger Lambelin’ |
| 7 | *R. rugosa* 'Pekingred' | 27 | *R. rugosa* ‘Tancity’ | 47 | *R. hybrida* ‘Royale’ | 67 | *R. hybrida* ‘Velvety Twilight’ |
| 8 | *R. rugosa* ‘Jingyou1’ | 28 | *R. hybrida* ‘Jinbian’ | 48 | *R. hybrida*‘Dream of Garden’ | 68 | *R. hybrida* ‘Nahéma’ |
| 9 | *R. rugosa* ‘Jingyou2’ | 29 | *R. hybrida* ‘Neixiang’ | 49 | *R. hybrida* ‘Autumn Rouge’ | 69 | *R. hybrida* ‘Libellula’ |
| 10 | *R. rugosa* 'Guo' | 30 | *R. hybrida* ‘Cepheus’ | 50 | *R. hybrida* ‘Haiku Romantika’ | 70 | *R. hybrida* ‘Crimson Glory’ |
| 11 | *R. rugosa* 'Pingyin8' | 31 | *R. hybrida* ‘White Ohara’ | 51 | *R. hybrida* ‘EdouardManet’ | 71 | *R.damascena.*Mill. |
| 12 | *R. rugosa* 'Pingyin11' | 32 | *R. hybrida* ‘My Beauty’ | 52 | *R. hybrida* ‘Aunt Margy's’ | 72 | *R. spp*. |
| 13 | *R. rugosa* 'Pingyin12' | 33 | *R. hybrida* ‘Red Eden Rose’ | 53 | *R. hybrida* ‘Fée Clochette’ | 73 | *R. gallica* |
| 14 | *R. rugosa* 'FanHua' | 34 | *R. hybrida* ‘Masora’ | 54 | *R. hybrida* ‘Blue Eden’ | 74 | *R. damascena.* albo |
| 15 | *R.rugosa* ‘Kushui’ | 35 | *R. hybrida* ‘Mon Coeur’ | 55 | *R. hybrida* ‘Accademia’ | 75 | *R. davurica* Pall. |
| 16 | *R. rugosa* ‘Mici’ | 36 | *R. hybrida* ‘Stephanie Baronlin zu Guttenberg’ | 56 | *R. hybrida* ‘Olivia’ | 76 | *R. centifolia* |
| 17 | *R. rugosa* ‘Hanxiang’ | 37 | *R. hybrida* ‘WiYaKo’ | 57 | *R. hybrida* ‘Poetry Kordana’ | 77 | *R.* ‘Dianhong’ |
| 18 | *R. rugosa ‘*BaiZiZhi’ | 38 | *R. hybrida* ‘Falstaff’ | 58 | *R. hybrida* ‘Misaki’ |  |  |
| 19 | *R. rugosa* 'XihuⅡ' | 39 | *R. hybrida* ‘Yumao’ | 59 | *R. hybrida* ‘Bienvenue’ |  |  |
| 20 | *R. rugosa* 'XihuⅢ' | 40 | *R. hybrida* ‘Vesalius’ | 60 | *R. hybrida* ‘The Wedgwood’ |  |  |
