# Supplementary material for: Evaluation of Rosa germplasm resources and analysis of floral fragrance components in R. rugosa
Source: Front Plant Sci. 2022 Oct 12;13:1026763. doi: 10.3389/fpls.2022.1026763 (PMC9597504; doi:10.3389/fpls.2022.1026763)
Supplement: Supplementary file 13 [file Table_2.docx]

**Table S2 Field raw data of 27 *R. rugosa***

| Name/Data | F1 Single/double petals | F2 Flower petal number | F3 Flower diameter | F9 The number of branches | F10 Plant morphology | F11 Pedicel length | F12 Internode length | F13 Crown width | F14 Main stem thickness | F15 Flowering branch thickness | F16 Plant height | F17 Total number of thorns | F18 Shape of the lower part of the thorns | F19 Presence or absence of thorns on flowering branches | F20Presence or absence of bristles on flowering branches | F21 Presence or absence of thorns on flowering pedicels | F22 Presence or absence of bristles on flowering pedicels |
| --- | --- | --- | --- | --- | --- | --- | --- | --- | --- | --- | --- | --- | --- | --- | --- | --- | --- |
| *R. rugosa* 'albo-plena' | double | 25.89 | 6.12 | 6 | 5 | 2.12 | 1.76 | 77.67/69.44 | 0.8 | 0.39 | 93.56 | 15.89 | 3 | presence | presence | absence | absence |
| *R. rugosa* ‘Purple Branch’ | double | 24.22 | 6.47 | 7.22 | 1 | 3.87 | 2.15 | 75.67/65.44 | 1.39 | 0.6 | 120.33 | 1.56 | 1 | presence | absence | absence | presence |
| *R. rugosa* 'Hezeyang' | double | 34.78 | 6.44 | 7.56 | 1 | 3.69 | 3.34 | 80.56/83.78 | 0.9 | 0.5 | 120.44 | 16.33 | 2 | presence | absence | presence | absence |
| *R. rugosa* ‘FenZiZhi’ | double | 30.67 | 5.62 | 6.67 | 1 | 3.95 | 2.14 | 79.67/75.89 | 1.03 | 0.34 | 113.67 | 0 | 1 | absence | absence | presence | absence |
| *R. rugosa* 'ZhongYuan' | double | 51.22 | 6.54 | 8.33 | 3 | 6.14 | 5.89 | 80.56/74.67 | 0.75 | 0.24 | 158.56 | 5.33 | 2 | presence | absence | presence | absence |
| *R. Rugosa* ‘Yilanxiao’ | double | 38.67 | 6 | 6 | 3 | 4.29 | 1.77 | 67.11/50.00 | 0.66 | 0.3 | 128.78 | 6.11 | 4 | presence | presence | presence | absence |
| *R. rugosa* 'Pekingred' | single | 5 | 6.29 | 7.33 | 7 | 2.27 | 1.19 | 75.11/64.00 | 0.89 | 0.51 | 85.11 | 83.67 | 1 | presence | presence | presence | absence |
| *R. rugosa* ‘Jingyou1’ | double | 39 | 6.71 | 8 | 3 | 2.93 | 1.36 | 79.00/69.89 | 0.9 | 0.3 | 145.56 | 4.44 | 3 | presence | presence | absence | presence |
| *R. rugosa* ‘Jingyou2’ | double | 42.22 | 5.64 | 10.33 | 3 | 3.72 | 1.83 | 76.89/64.67 | 0.83 | 0.3 | 140.22 | 3 | 3 | presence | presence | presence | absence |
| *R. rugosa* 'Guo' | single | 5 | 3.81 | 6 | 5 | 1.41 | 1.07 | 69.33/64.89 | 0.56 | 0.25 | 108.67 | 5.67 | 4 | presence | absence | presence | absence |
| *R. rugosa* 'Pingyin8' | double | 17.44 | 7.36 | 5.67 | 1 | 1.88 | 1.41 | 64.33/59.78 | 0.78 | 0.4 | 120.00 | 12.56 | 3 | presence | presence | absence | presence |
| *R. rugosa* 'Pingyin11' | double | 14 | 5.85 | 7.11 | 5 | 2.24 | 1.36 | 69.56/71.00 | 0.98 | 0.45 | 111.89 | 9.56 | 3 | presence | presence | absence | presence |
| *R. rugosa* 'Pingyin12' | double | 27.33 | 6.44 | 10.89 | 3 | 1.69 | 1.82 | 81.00/85.33 | 0.84 | 0.3 | 150.22 | 296.11 | 1 | presence | absence | presence | absence |
| *R. rugosa* 'FanHua' | single | 12.56 | 6.63 | 9.78 | 1 | 1.79 | 1.55 | 67.89/80.22 | 1.16 | 0.36 | 139.22 | 22.67 | 1 | presence | absence | absence | absence |
| *R.sertate×R.rugosa* | single | 16 | 6.03 | 7.22 | 7 | 1.66 | 1.77 | 61.11/57.00 | 0.68 | 0.4 | 92.78 | 0 | 0 | absence | absence | absence | presence |
| *R. rugosa* ‘Mici’ | double | 36.56 | 7.42 | 8.89 | 3 | 5.2 | 1.68 | 68.78/59.89 | 0.96 | 0.4 | 151.44 | 8.56 | 4 | presence | presence | absence | presence |
| *R. rugosa* ‘Hanxiang’ | double | 42.11 | 6.14 | 6.78 | 3 | 2.16 | 1.75 | 69.11/79.67 | 0.85 | 0.35 | 148.78 | 4.89 | 4 | presence | absence | absence | presence |
| *R. rugosa ‘*BaiZiZhi’ | double | 21.78 | 6.19 | 6.89 | 3 | 2.17 | 2.14 | 78.67/89.56 | 0.91 | 0.59 | 167.44 | 3.22 | 1 | presence | absence | absence | absence |
| *R. rugosa* 'XihuⅡ' | double | 24.33 | 6.21 | 11.33 | 3 | 1.92 | 1.49 | 87.44/85.44 | 0.77 | 0.5 | 112.89 | 36 | 2 | presence | presence | absence | presence |
| *R. rugosa* 'XihuⅢ' | double | 15.89 | 7.2 | 6.89 | 3 | 2.05 | 1.47 | 89.00/85.22 | 0.86 | 0.46 | 141.33 | 9.67 | 1 | presence | absence | absence | presence |
| *R. rugosa ‘*LiangYeHong’ | double | 25 | 7.61 | 15.22 | 3 | 3.51 | 1.18 | 55.44/70.00 | 1.04 | 0.51 | 170.67 | 28.67 | 1 | presence | absence | absence | absence |
| *R. rugosa* ‘DaGuo’ | double | 16.67 | 7.26 | 8.67 | 5 | 1.96 | 3.99 | 59.89/78.67 | 0.82 | 0.41 | 160.11 | 5.56 | 1 | presence | absence | absence | presence |
| *R. rugosa* 'GaoHong' | double | 19.22 | 5.68 | 8.89 | 1 | 3.94 | 3.47 | 67.89/59.78 | 1.64 | 0.6 | 230.56 | 14.44 | 0 | absence | absence | presence | absence |
| *R. rugosa* 'Pekingwhite' | single | 5 | 5.92 | 12 | 3 | 1.73 | 1.65 | 80.33/84.44 | 1.28 | 0.55 | 115.33 | 70.33 | 1 | presence | presence | absence | absence |
| *R. rugosa* ‘Lufthansa’ | double | 38.44 | 6.66 | 6 | 7 | 2.51 | 1.1 | 83.67/69.00 | 0.87 | 0.4 | 62.89 | 6.89 | 2 | presence | absence | absence | absence |
| *R. rugosa* 'TianEHuang' | double | 31.11 | 5.73 | 7.22 | 5 | 2.36 | 3.17 | 60.00/75.56 | 0.79 | 0.3 | 121.11 | 9.67 | 3 | presence | absence | presence | absence |
| *R. rugosa* ‘Tancity’ | double | 23.78 | 7.07 | 5.78 | 3 | 4.07 | 1.29 | 89.78/75.22 | 1.1 | 0.52 | 120.22 | 7.11 | 3 | presence | absence | presence | absence |

Note: The unit length is cm. It is repeated nine times, and the average value is taken.
