# Supplementary material for: Evaluation of Rosa germplasm resources and analysis of floral fragrance components in R. rugosa
Source: Front Plant Sci. 2022 Oct 12;13:1026763. doi: 10.3389/fpls.2022.1026763 (PMC9597504; doi:10.3389/fpls.2022.1026763)
Supplement: Supplementary file 14 [file Table_3.docx]

**Table S3 Raw field data for 43 scented *R. hybrida* cultivars and seven aromatic *Rosa* species.**

| Name/Data | Single/double petals | Flower petal number | Flower diameter | Pedicel length | Internode length | Total number of thorns | Presence or absence of thorns on flowering branches | | Presence or absence of bristles on flowering branches | | Presence or absence of thorns on flowering pedicels | Presence or absence of bristles on flowering pedicels |
| --- | --- | --- | --- | --- | --- | --- | --- | --- | --- | --- | --- | --- |
| *R. multiflora* Thunb.*Hybrida* | double | 58.46 | 2.70 | 3.20 | 2.69 | 4.67 | | presence | | absence | presence | absence |
| *R. hybrida* ‘Neixiang’ | double | 85.58 | 5.80 | 3.30 | 3.07 | 8.33 | | presence | | presence | absence | presence |
| *R. hybrida* ‘Cepheus’ | double | 145.62 | 5.78 | 6.07 | 2.23 | 5.52 | | presence | | absence | presence | absence |
| *R. hybrida* ‘White Ohara’ | double | 107.55 | 6.88 | 3.84 | 5.58 | 3.33 | | presence | | absence | presence | absence |
| *R. hybrida* ‘My Beauty’ | double | 47.64 | 5.25 | 2.35 | 2.97 | 2.00 | | presence | | absence | presence | absence |
| *R. hybrida* ‘Red Eden Rose’ | double | 131.00 | 6.60 | 3.75 | 3.50 | 10.00 | | presence | | absence | absence | presence |
| *R. hybrida* ‘Masora’ | double | 142.23 | 6.30 | 4.11 | 3.40 | 3.33 | | absence | | absence | absence | presence |
| *R. hybrida* ‘Mon Coeur’ | double | 148.00 | 4.80 | 3.70 | 4.90 | 1.00 | | presence | | absence | absence | presence |
| *R. hybrida* ‘Stephanie Baronlin zu Guttenberg’ | double | 98.50 | 5.40 | 3.50 | 2.78 | 4.33 | | presence | | absence | presence | absence |
| *R. hybrida* ‘WiYaKo’ | double | 61.20 | 6.70 | 4.52 | 2.43 | 0.00 | | absence | | absence | absence | absence |
| *R. hybrida* ‘Falstaff’ | double | 118.40 | 6.50 | 6.51 | 4.04 | 27.33 | | presence | | presence | absence | presence |
| *R. hybrida* ‘Yumao’ | double | 135.66 | 7.12 | 4.30 | 2.96 | 3.67 | | presence | | absence | absence | presence |
| *R. hybrida* ‘Vesalius’ | double | 139.50 | 5.20 | 4.11 | 2.54 | 0.00 | | absence | | absence | absence | absence |
| *R, hybrida* ‘Spirit of Freedom’ | double | 214.20 | 6.50 | 4.52 | 3.53 | 31.00 | | presence | | presence | absence | presence |
| *R, hybrida* ‘Swallow’ | double | 135.00 | 7.40 | 5.12 | 2.55 | 21.00 | | presence | | presence | presence | absence |
| *R. hybrida* ‘William Morris’ | double | 95.21 | 6.20 | 3.75 | 3.80 | 7.33 | | presence | | absence | absence | absence |
| *R. hybrida* ‘Sweet Chariot’ | double | 88.24 | 3.50 | 1.95 | 2.63 | 4.10 | | presence | | absence | absence | absence |
| *R. hybrida* ‘Golden Celebration’ | double | 138.35 | 6.20 | 4.10 | 3.28 | 2.67 | | presence | | absence | absence | absence |
| *R. hybrida* ‘Odysseia’ | double | 136.22 | 6.10 | 3.80 | 5.55 | 0.00 | | absence | | absence | absence | presence |
| *R. hybrida* ‘Royale’ | double | 35.32 | 8.90 | 7.40 | 4.43 | 10.00 | | presence | | absence | absence | presence |
| *R. hybrida*‘Dream of Garden’ | double | 58.00 | 7.20 | 6.33 | 4.30 | 6.00 | | presence | | absence | absence | presence |
| *R. hybrida* ‘Autumn Rouge’ | double | 121.56 | 5.50 | 4.60 | 3.84 | 9.33 | | presence | | absence | presence | absence |
| *R. hybrida* ‘Haiku Romantika’ | double | 130.20 | 7.40 | 4.73 | 3.42 | 2.67 | | presence | | absence | absence | absence |
| *R. hybrida* ‘EdouardManet’ | double | 87.78 | 6.87 | 3.33 | 3.59 | 1.20 | | presence | | absence | absence | absence |
| *R. hybrida* ‘Aunt Margy's’ | double | 96.89 | 4.07 | 5.10 | 3.81 | 3.00 | | presence | | absence | presence | absence |
| *R. hybrida* ‘Fée Clochette’ | double | 131.89 | 5.82 | 6.36 | 2.19 | 0.00 | | absence | | presence | presence | absence |
| *R. hybrida* ‘Blue Eden’ | double | 29.56 | 8.86 | 5.01 | 3.30 | 6.50 | | presence | | absence | presence | absence |
| *R. hybrida* ‘Accademia’ | double | 150.89 | 7.13 | 6.22 | 2.40 | 0.00 | | absence | | presence | absence | absence |
| *R. hybrida* ‘Olivia’ | double | 52.67 | 6.84 | 6.44 | 3.30 | 7.00 | | presence | | absence | presence | absence |
| *R. hybrida* ‘Poetry Kordana’ | double | 153.56 | 5.57 | 4.01 | 3.66 | 2.20 | | presence | | absence | absence | absence |
| *R. hybrida* ‘Misaki’ | double | 108.23 | 7.76 | 6.67 | 4.10 | 0.00 | | absence | | absence | presence | absence |
| *R. hybrida* ‘Bienvenue’ | double | 97.44 | 6.21 | 3.89 | 2.54 | 5.00 | | presence | | absence | absence | absence |
| *R. hybrida* ‘The Wedgwood’ | double | 79.11 | 6.83 | 5.44 | 4.79 | 12.33 | | presence | | absence | presence | absence |
| *R. hybrida* ‘Kayla’ | double | 66.38 | 6.38 | 5.14 | 2.72 | 0.00 | | absence | | presence | presence | absence |
| *R. hybrida* ‘Crown Princess Margareta’ | double | 101.20 | 7.52 | 5.50 | 3.04 | 0.00 | | absence | | absence | absence | presence |
| *R. hybrida* ‘Geoff Hamilton ’ | double | 218.78 | 5.49 | 3.33 | 2.34 | 0.00 | | absence | | absence | presence | absence |
| *R. hybrida* ‘Yua’ | double | 156.25 | 5.39 | 6.36 | 3.12 | 0.00 | | absence | | absence | absence | absence |
| *R. hybrida* ‘Paul Neyron’ | double | 116.44 | 7.52 | 5.37 | 3.15 | 0.00 | | absence | | absence | absence | absence |
| *R. hybrida* ‘Roger Lambelin’ | double | 29.11 | 5.48 | 2.92 | 2.86 | 6.67 | | presence | | presence | absence | absence |
| *R. hybrida* ‘Velvety Twilight’ | double | 71.89 | 6.38 | 4.22 | 3.16 | 23.67 | | presence | | absence | absence | absence |
| *R. hybrida* ‘Nahéma’ | double | 147.22 | 6.11 | 6.08 | 4.50 | 0.00 | | absence | | absence | presence | absence |
| *R. hybrida* ‘Libellula’ | double | 35.67 | 6.23 | 2.83 | 3.23 | 3.21 | | presence | | absence | absence | absence |
| *R. hybrida* cv.Crimson Glory | double | 23.78 | 6.22 | 5.72 | 2.72 | 22.20 | | presence | | absence | presence | absence |
| *R.damascna* | double | 38.44 | 5.66 | 3.29 | 2.91 | 55.22 | | presence | | presence | presence | absence |
| *R.* damascena Mill. | Single | 5.00 | 1.94 | 1.89 | 2.47 | 8.67 | | presence | | absence | absence | absence |
| *R. gallica* | double | 50.00 | 6.09 | 4.46 | 2.12 | 5.22 | | absence | | absence | presence | absence |
| *R. damascena* albo | double | 51.44 | 5.72 | 3.28 | 2.87 | 2.78 | | presence | | absence | presence | absence |
| *R. davurica* Pall. | Single | 5.00 | 4.87 | 1.89 | 1.82 | 0.00 | | absence | | absence | presence | absence |
| *R. centifolia* | double | 36.00 | 6.27 | 3.50 | 2.37 | 0.00 | | absence | | presence | presence | absence |
| *R.gallica* var. lunnanum ruber | double | 87.00 | 5.95 | 2.61 | 2.66 | 12.89 | | presence | | absence | absence | absence |

Note: unit of length is cm, which is repeated for nine times, and the average value is taken.
