# Supplementary material for: Evaluation of Rosa germplasm resources and analysis of floral fragrance components in R. rugosa
Source: Front Plant Sci. 2022 Oct 12;13:1026763. doi: 10.3389/fpls.2022.1026763 (PMC9597504; doi:10.3389/fpls.2022.1026763)
Supplement: Supplementary file 15 [file Table_4.docx]

**Table S4 The 16 main metabolite content of 27 *R. rugosa***

| Compounds | Class | ‘Guo’ | ‘Hanxiang’ | ‘Baizizhi’ | 'Pekingwhite' | ‘FenZiZhi’ | ‘Tancity’ | ‘Mici’ | ‘Jingyou2’ | 'Pingyin11' | 'Pingyin12' | 'FanHua' | R.sertate×R.rugosa | ‘Jingyou1’ | ‘Purple Branch’ |
| --- | --- | --- | --- | --- | --- | --- | --- | --- | --- | --- | --- | --- | --- | --- | --- |
| 1 | terpene | 0.21 | 0.08 | 0.19 | 0.13 | 0.04 | 0.05 | 0.13 | 0.14 | 0.20 | 0.20 | 0.08 | 0.06 | 0.14 | 0.22 |
| 2 | terpene | 0.15 | 0.02 | 0.02 | 0.02 | 0.02 | 0.02 | 0.02 | 0.02 | 0.03 | 0.02 | 0.05 | 0.03 | 0.02 | 0.03 |
| 3 | terpene | 3.43 | 3.21 | 1.09 | 0.34 | 1.04 | 2.46 | 1.90 | 1.99 | 1.32 | 2.31 | 1.59 | 1.31 | 2.05 | 2.01 |
| 4 | ester | 0.11 | 0.03 | 0.02 | 0.01 | 0.01 | 0.02 | 0.03 | 0.03 | 0.03 | 0.04 | 0.04 | 0.02 | 0.03 | 0.07 |
| 5 | terpene | 2.33 | 1.22 | 1.22 | 1.20 | 1.21 | 1.21 | 1.21 | 1.23 | 1.26 | 1.19 | 1.41 | 1.26 | 1.21 | 1.23 |
| 6 | terpene | 0.69 | 0.37 | 0.20 | 0.23 | 0.15 | 0.76 | 0.61 | 0.45 | 0.88 | 0.57 | 0.25 | 0.44 | 0.56 | 0.01 |
| 7 | ester | 0.28 | 0.20 | 0.17 | 0.02 | 0.01 | 0.03 | 0.19 | 0.21 | 0.12 | 0.30 | 0.04 | 0.03 | 0.21 | 0.16 |
| 8 | ester | 0.00 | 0.03 | 0.04 | 0.09 | 0.02 | 0.02 | 0.06 | 0.07 | 0.01 | 0.14 | 0.07 | 0.00 | 0.05 | 0.03 |
| 9 | terpene | 0.12 | 0.10 | 0.03 | 0.02 | 0.02 | 0.06 | 0.09 | 0.07 | 0.11 | 0.07 | 0.07 | 0.07 | 0.07 | 0.02 |
| 10 | terpene | 5.43 | 12.39 | 4.92 | 0.98 | 1.73 | 3.72 | 7.17 | 8.18 | 2.31 | 6.27 | 1.29 | 1.66 | 7.67 | 3.40 |
| 11 | phenol | 0.69 | 0.29 | 0.78 | 0.19 | 0.63 | 0.27 | 0.33 | 0.15 | 0.62 | 0.82 | 0.44 | 0.25 | 0.27 | 0.45 |
| 12 | ester | 0.80 | 0.25 | 0.06 | 0.09 | 0.02 | 0.04 | 0.27 | 0.27 | 0.37 | 0.37 | 0.48 | 0.12 | 0.28 | 0.39 |
| 13 | terpene | 0.99 | 1.36 | 0.27 | 0.22 | 0.20 | 0.58 | 0.56 | 0.64 | 0.62 | 0.68 | 0.73 | 0.69 | 0.69 | 0.47 |
| 14 | phenol | 0.25 | 0.13 | 0.44 | 0.15 | 0.08 | 0.20 | 0.15 | 0.07 | 0.07 | 0.27 | 0.07 | 0.02 | 0.12 | 0.16 |
| 15 | alcohol | 0.00 | 14.35 | 23.17 | 10.38 | 13.83 | 12.29 | 9.60 | 12.61 | 3.34 | 15.83 | 14.84 | 0.85 | 12.63 | 17.74 |
| 16 | heterocyclic compound | 0.04 | 0.03 | 0.03 | 0.03 | 0.03 | 0.03 | 0.03 | 0.03 | 0.03 | 0.03 | 0.03 | 0.03 | 0.03 | 0.03 |

| Compounds | Class | 'Hezeyang' | 'ZhongYuan' | ‘Yilanxiao’ | 'TianEHuang' | 'Pekingred' | 'Pingyin8' | 'XihuⅡ' | 'XihuⅢ' | ‘LiangYeHong’ | ‘DaGuo’ | 'GaoHong' | 'albo-plena' | ‘Lufthansa’ |
| --- | --- | --- | --- | --- | --- | --- | --- | --- | --- | --- | --- | --- | --- | --- |
| 1 | terpene | 0.05 | 0.03 | 0.22 | 0.04 | 0.15 | 0.07 | 0.23 | 0.17 | 0.17 | 0.07 | 0.03 | 0.11 | 0.18 |
| 2 | terpene | 0.02 | 0.02 | 0.02 | 0.02 | 0.02 | 0.04 | 0.02 | 0.03 | 0.02 | 0.04 | 0.02 | 0.02 | 0.03 |
| 3 | terpene | 3.53 | 0.71 | 1.69 | 0.20 | 2.23 | 1.78 | 1.66 | 1.32 | 1.17 | 1.87 | 0.64 | 2.61 | 2.27 |
| 4 | ester | 0.11 | 0.01 | 0.03 | 0.00 | 0.01 | 0.02 | 0.07 | 0.03 | 0.02 | 0.02 | 0.01 | 0.02 | 0.07 |
| 5 | terpene | 1.22 | 1.19 | 1.23 | 1.19 | 1.17 | 1.34 | 1.22 | 1.25 | 1.19 | 1.34 | 1.20 | 1.20 | 1.28 |
| 6 | terpene | 0.01 | 0.10 | 0.34 | 0.00 | 0.50 | 0.22 | 0.21 | 0.31 | 0.33 | 0.22 | 0.14 | 0.31 | 0.36 |
| 7 | ester | 0.08 | 0.06 | 0.15 | 0.01 | 0.02 | 0.08 | 0.14 | 0.12 | 0.09 | 0.08 | 0.00 | 0.06 | 0.12 |
| 8 | ester | 0.07 | 0.32 | 0.06 | 0.09 | 0.02 | 0.05 | 0.12 | 0.01 | 0.13 | 0.08 | 0.03 | 0.03 | 0.01 |
| 9 | terpene | 0.03 | 0.01 | 0.05 | 0.00 | 0.05 | 0.07 | 0.04 | 0.05 | 0.05 | 0.06 | 0.05 | 0.05 | 0.06 |
| 10 | terpene | 1.47 | 1.46 | 5.53 | 0.00 | 1.84 | 4.35 | 2.10 | 2.63 | 4.65 | 4.05 | 1.21 | 3.61 | 1.60 |
| 11 | phenol | 0.05 | 0.05 | 0.25 | 0.60 | 0.40 | 0.52 | 0.66 | 0.49 | 0.14 | 0.66 | 0.16 | 0.43 | 0.35 |
| 12 | ester | 0.35 | 0.03 | 0.23 | 0.01 | 0.02 | 0.09 | 0.51 | 0.35 | 0.08 | 0.12 | 0.01 | 0.14 | 0.40 |
| 13 | terpene | 0.76 | 0.19 | 0.53 | 0.09 | 0.25 | 0.68 | 0.41 | 0.62 | 0.25 | 0.68 | 0.13 | 0.56 | 0.37 |
| 14 | phenol | 0.00 | 0.01 | 0.21 | 0.00 | 0.01 | 0.13 | 0.20 | 0.10 | 0.10 | 0.11 | 0.03 | 0.19 | 0.11 |
| 15 | alcohol | 5.73 | 10.12 | 12.73 | 24.31 | 3.61 | 17.13 | 16.22 | 3.28 | 16.27 | 15.78 | 10.29 | 16.21 | 4.42 |
| 16 | heterocyclic compound | 0.03 | 0.03 | 0.03 | 0.03 | 0.03 | 0.03 | 0.03 | 0.03 | 0.03 | 0.03 | 0.03 | 0.03 | 0.03 |

注：单位为μg/g：1α-法尼烯；2橙花叔醇；3橙花醇；4金合欢醇；5玫瑰醚；6香茅醛；7香茅醇；8芳樟醇；9乙酸橙花酯；10乙酸香茅酯；11乙酸苯乙酯；12乙酸香叶酯；13丁香酚；14甲基丁香酚；15苯乙醇；16反式-芳樟醇氧化物
