# Supplementary material for: Evaluation of Rosa germplasm resources and analysis of floral fragrance components in R. rugosa
Source: Front Plant Sci. 2022 Oct 12;13:1026763. doi: 10.3389/fpls.2022.1026763 (PMC9597504; doi:10.3389/fpls.2022.1026763)
Supplement: Supplementary file 16 [file Table_5.docx]

**Table S5 The five main metabolite content of 27 *R. rugosa***

| Compounds | Class | ‘Guo’ | ‘Hanxiang’ | ‘Baizizhi’ | 'Pekingwhite' | ‘FenZiZhi’ | ‘Tancity’ | ‘Mici’ | ‘Jingyou2’ | 'Pingyin11' | 'Pingyin12' | 'FanHua' | *R.sertate×R.rugosa* | ‘Jingyou1’ | ‘PurpleBranch’ |
| --- | --- | --- | --- | --- | --- | --- | --- | --- | --- | --- | --- | --- | --- | --- | --- |
| F4 | Ester | 0.28 | 0.20 | 0.17 | 0.02 | 0.01 | 0.03 | 0.19 | 0.21 | 0.12 | 0.30 | 0.04 | 0.03 | 0.21 | 0.16 |
| F5 | Alcohol | 0.00 | 14.35 | 23.17 | 10.38 | 13.83 | 12.29 | 9.60 | 12.61 | 3.34 | 15.83 | 14.84 | 0.85 | 12.63 | 17.74 |
| F6 | Ester | 0.11 | 0.03 | 0.02 | 0.01 | 0.01 | 0.02 | 0.03 | 0.03 | 0.03 | 0.04 | 0.04 | 0.02 | 0.03 | 0.07 |
| F7 | Terpenes | 3.43 | 3.21 | 1.09 | 0.34 | 1.04 | 2.46 | 1.90 | 1.99 | 1.32 | 2.31 | 1.59 | 1.31 | 2.05 | 2.01 |
| F8 | Terpenes | 2.33 | 1.22 | 1.22 | 1.20 | 1.21 | 1.21 | 1.21 | 1.23 | 1.26 | 1.19 | 1.41 | 1.26 | 1.21 | 1.23 |

| Compounds | Class | 'Hezeyang' | 'ZhongYuan' | ‘Yilanxiao’ | 'TianEHuang' | 'Pekingred' | 'Pingyin8' | 'XihuⅡ' | 'XihuⅢ' | ‘LiangYeHong’ | ‘DaGuo’ | 'GaoHong' | 'albo-plena' | ‘Lufthansa’ |
| --- | --- | --- | --- | --- | --- | --- | --- | --- | --- | --- | --- | --- | --- | --- |
| F4 | Ester | 0.08 | 0.06 | 0.15 | 0.01 | 0.02 | 0.08 | 0.14 | 0.12 | 0.09 | 0.08 | 0.00 | 3.61 | 0.12 |
| F5 | Alcohol | 5.73 | 10.12 | 12.73 | 24.31 | 3.61 | 17.13 | 16.22 | 3.28 | 16.27 | 15.78 | 10.29 | 16.21 | 4.42 |
| F6 | Ester | 0.11 | 0.01 | 0.03 | 0.00 | 0.01 | 0.02 | 0.07 | 0.03 | 0.02 | 0.02 | 0.01 | 1.20 | 0.07 |
| F7 | Terpenes | 3.53 | 0.71 | 1.69 | 0.20 | 2.23 | 1.78 | 1.66 | 1.32 | 1.17 | 1.87 | 0.64 | 2.61 | 2.27 |
| F8 | Terpenes | 1.22 | 1.19 | 1.23 | 1.19 | 1.17 | 1.34 | 1.22 | 1.25 | 1.19 | 1.34 | 1.20 | 0.31 | 1.28 |

Note: Unit is μg/g. F4 Citronellol, F5 Phenethyl alcohol, F6 Farnesol, F7 Nerol, F8 Rose oxide. Average of three replicates.
