# Supplementary material for: Evaluation of Rosa germplasm resources and analysis of floral fragrance components in R. rugosa
Source: Front Plant Sci. 2022 Oct 12;13:1026763. doi: 10.3389/fpls.2022.1026763 (PMC9597504; doi:10.3389/fpls.2022.1026763)
Supplement: Supplementary file 17 [file Table_6.docx]

**Table S6 Standard of evaluation index scores for *Rosa rugosa***

| Score | | | | | |
| --- | --- | --- | --- | --- | --- |
|  | 5 | 4 | 3 | 2 | 1 |
| Single or double petals | \ | \ | Double petals | \ | Single petal |
| The number of petals | ≥50 | 30-50 | 20-30 | 20-5 | <5 |
| Flower diameter（cm） | ≥7 | 6.5-7 | 6-6.5 | 5-6 | <5 |
| Citronellol（μg/g） | ≥5 | 3-5 | 2-3 | 1-2 | 0 |
| Phenylethyl alcohol（μg/g） | ≥15 | 11-15 | 9-11 | 1-9 | 0-1 |
| Farnesol（μg/g） | ≥1.5 | 1.2-1.5 | <1.2 | - | - |
| Nerol（μg/g） | ≥3 | 2-3 | 1.5-2 | 1-1.5 | <1 |
| Rose oxide（μg/g） | ≥0.5 | 0.3-0.5 | 0.2-0.3 | 0.1-0.2 | 0 |
| The number of branches | ≥9 | 8-9 | 7-8 | 6-7 | <6 |
| Plant morphology | 1 | 3 | 5 | 7 | 9 |
| The length of the pedicel（cm） | ≥6 | 5-6 | 3-5 | 2-3 | 1-2 |
| The length of the internodes（cm） | <2 | 2-2.5 | 2.5-3.5 | 3.5-4.5 | ≥4.5 |
| Crown width（cm） | ≥83 | 78-83 | 75-78 | 69-75 | <69 |
| The thickness of the main stem（cm） | ≥1 | 0.9-1 | 0.8-0.9 | 0.7-0.8 | <0.7 |
| The thickness of the flowering branches（cm） | ≥0.5 | 0.4-0.5 | 0.3-0.4 | 0.3 | <0.3 |
| The height of the whole plant（cm） | 60-150  （Most suitable for picking） | 150-200 | ≥200 | 30-50 | <30 |
| The number of prickles | 0 | 1-15 | 15-50 | 50-100 | ≥100 |
| The shape of the lower part of the prickles | 0（Don’t have） | 4 | 3 | 2 | 1 |
| With or without flower branch pricks | \ | \ | absence | \ | presence |
| With or without flower branch bristles | \ | \ | absence | \ | presence |
| With or without pedicel pricks | \ | \ | absence | \ | presence |
| With or without pedicel bristles | \ | \ | absence | \ | presence |
