# Supplementary material for: Evaluation of Rosa germplasm resources and analysis of floral fragrance components in R. rugosa
Source: Front Plant Sci. 2022 Oct 12;13:1026763. doi: 10.3389/fpls.2022.1026763 (PMC9597504; doi:10.3389/fpls.2022.1026763)
Supplement: Supplementary file 18 [file Table_7.docx]

**Table S7 Total difference metabolite trend content**

| Sub Class | Compounds | Class | BZ1 | BZ3 | BZ5 | BZ7 | HX1 | HX3 | HX5 | HX7 | GM1 | GM3 | GM5 | GM7 |
| --- | --- | --- | --- | --- | --- | --- | --- | --- | --- | --- | --- | --- | --- | --- |
| 1 | 1,3-Cyclohexadiene-1-carboxaldehyde, 2,6,6-trimethyl- | Terpenes | 5.37E+03 | 5.01E+03 | 4.44E+03 | 8.56E+03 | 2.02E+03 | 1.00E+00 | 3.76E+03 | 6.22E+03 | 1.69E+03 | 6.06E+03 | 6.17E+03 | 7.59E+03 |
| 1 | 1-Heptanol | Alcohol | 9.86E+04 | 6.15E+04 | 1.44E+05 | 2.99E+05 | 5.80E+04 | 2.18E+04 | 2.86E+04 | 2.03E+05 | 2.15E+05 | 2.82E+05 | 2.62E+05 | 8.42E+05 |
| 1 | Eugenol | Phenol | 1.59E+06 | 3.54E+06 | 1.09E+07 | 1.24E+07 | 2.10E+06 | 2.35E+06 | 4.05E+06 | 5.98E+06 | 3.82E+06 | 4.07E+06 | 1.03E+07 | 2.41E+07 |
| 1 | 2,6-Octadien-1-ol, 3,7-dimethyl-, acetate, (Z)- | Ester | 1.00E+00 | 1.15E+05 | 8.84E+04 | 2.60E+04 | 1.00E+00 | 1.00E+00 | 1.35E+05 | 3.11E+05 | 8.45E+03 | 3.24E+05 | 8.92E+05 | 8.88E+05 |
| 1 | 1H-Inden-5-ol, 2,3-dihydro- | Phenol | 1.00E+00 | 1.15E+04 | 2.74E+04 | 2.30E+04 | 1.00E+00 | 1.00E+00 | 8.32E+03 | 1.27E+04 | 1.54E+04 | 1.14E+04 | 2.63E+04 | 4.52E+04 |
| 1 | Naphthalene, 2-methyl- | Aromatics | 4.34E+03 | 1.79E+04 | 1.97E+04 | 2.88E+04 | 6.78E+03 | 6.59E+03 | 8.04E+03 | 1.69E+04 | 1.11E+04 | 1.31E+04 | 2.13E+04 | 1.87E+04 |
| 1 | Butanoic acid, 2-pentenyl ester, (Z)- | Ester | 9.88E+03 | 2.16E+04 | 5.69E+04 | 6.45E+04 | 8.54E+03 | 6.73E+03 | 1.22E+04 | 4.03E+04 | 2.59E+04 | 3.01E+04 | 6.21E+04 | 1.13E+05 |
| 1 | Naphthalene, 1,2-dihydro-4,5,7-trimethyl- | Aromatics | 1.23E+04 | 8.12E+03 | 2.33E+04 | 2.44E+04 | 9.31E+03 | 2.36E+04 | 1.51E+04 | 1.80E+04 | 1.14E+04 | 2.49E+04 | 2.66E+04 | 3.85E+04 |
| 2 | Dimethyl sulfone | Sulfide | 5.06E+04 | 3.43E+04 | 2.29E+04 | 2.86E+04 | 6.42E+04 | 1.34E+04 | 7.17E+04 | 8.85E+04 | 1.94E+04 | 4.33E+04 | 6.90E+04 | 3.23E+04 |
| 2 | 3-Methyl-2-(2-methyl-2-butenyl)-furan | Heterocyclic compound | 1.00E+04 | 7.20E+03 | 3.40E+03 | 7.53E+03 | 8.67E+03 | 7.74E+02 | 1.97E+04 | 2.38E+04 | 1.42E+03 | 8.84E+03 | 1.29E+04 | 1.02E+04 |
| 2 | 2,6-Octadienal, 3,7-dimethyl-, (E)- | Terpenes | 2.48E+06 | 2.09E+06 | 9.68E+05 | 1.86E+06 | 2.21E+06 | 2.73E+05 | 5.33E+06 | 4.27E+06 | 4.16E+05 | 2.76E+06 | 3.72E+06 | 2.89E+06 |
| 2 | Ethanone, 1-(1-ethyl-3-methyl-1H-pyrazol-4-yl)- | Ketone | 7.84E+05 | 6.72E+05 | 3.10E+05 | 5.91E+05 | 6.98E+05 | 8.17E+04 | 1.66E+06 | 1.46E+06 | 1.25E+05 | 9.12E+05 | 1.26E+06 | 9.75E+05 |
| 2 | Ethanone, 1-(1,4-dimethyl-3-cyclohexen-1-yl)- | Terpenes | 3.01E+05 | 2.76E+05 | 1.56E+05 | 2.88E+05 | 2.73E+05 | 3.31E+04 | 6.25E+05 | 7.31E+05 | 5.02E+04 | 3.54E+05 | 5.40E+05 | 4.16E+05 |
| 2 | 6-Octenal, 7-methyl-3-methylene- | Aldehyde | 8.11E+04 | 6.51E+04 | 2.91E+04 | 5.45E+04 | 1.35E+05 | 1.62E+04 | 2.07E+05 | 1.90E+05 | 2.55E+04 | 1.04E+05 | 1.31E+05 | 9.32E+04 |
| 2 | Isogeranial | Amine | 3.69E+04 | 3.10E+04 | 1.47E+04 | 3.02E+04 | 3.33E+04 | 2.42E+03 | 1.16E+05 | 1.14E+05 | 4.38E+03 | 4.55E+04 | 7.16E+04 | 4.80E+04 |
| 2 | 5-Isopropenyl-2-methylcyclopent-1-enecarboxaldehyde | Aldehyde | 4.28E+04 | 2.15E+04 | 9.83E+03 | 2.23E+04 | 2.54E+04 | 2.20E+03 | 5.58E+04 | 6.09E+04 | 3.76E+03 | 2.39E+04 | 3.12E+04 | 2.34E+04 |
| 2 | Benzene, pentamethyl- | Aromatics | 2.69E+04 | 3.20E+04 | 2.27E+04 | 4.02E+04 | 2.62E+04 | 7.64E+03 | 4.66E+04 | 7.23E+04 | 1.43E+04 | 3.17E+04 | 4.05E+04 | 3.70E+04 |
| 2 | L-.alpha.-Terpineol | Terpenes | 9.18E+05 | 6.34E+05 | 2.81E+05 | 2.96E+05 | 1.04E+06 | 3.89E+05 | 1.26E+06 | 1.41E+06 | 2.16E+05 | 5.72E+05 | 9.88E+05 | 5.01E+05 |
| 2 | 6-Octen-1-ol, 3,7-dimethyl-, formate | Terpenes | 2.48E+06 | 2.15E+06 | 1.01E+06 | 1.88E+06 | 2.21E+06 | 2.74E+05 | 5.31E+06 | 4.46E+06 | 4.20E+05 | 2.91E+06 | 4.07E+06 | 2.99E+06 |
| 2 | 3,4,5-Trifluorobenzyl alcohol, 2-methylpropyl ether | Alcohol | 1.59E+04 | 9.36E+03 | 4.87E+03 | 1.18E+04 | 1.03E+04 | 1.82E+03 | 1.60E+04 | 2.06E+04 | 3.31E+03 | 8.00E+03 | 1.05E+04 | 1.04E+04 |
| 2 | 4,7,7-Trimethylbicyclo[4.1.0]hept-3-en-2-one | Ketone | 1.24E+04 | 1.56E+04 | 7.78E+03 | 1.67E+04 | 1.37E+04 | 1.00E+00 | 4.31E+04 | 6.23E+04 | 1.00E+00 | 1.99E+04 | 3.60E+04 | 2.63E+04 |
| 3 | trans-Linalool oxide (furanoid) | Heterocyclic compound | 3.55E+04 | 1.31E+04 | 8.81E+03 | 4.08E+04 | 6.69E+04 | 1.46E+04 | 1.44E+04 | 6.66E+04 | 4.86E+04 | 2.39E+04 | 8.84E+04 | 4.21E+04 |
| 3 | 2,6-Octadienal, 3,7-dimethyl-, (Z)- | Terpenes | 2.57E+05 | 2.52E+05 | 1.14E+05 | 2.10E+05 | 2.56E+05 | 1.59E+04 | 2.55E+05 | 2.77E+05 | 2.24E+04 | 3.66E+05 | 5.67E+05 | 3.60E+05 |
| 3 | cis-.beta.-Farnesene | Terpenes | 7.74E+02 | 2.68E+04 | 3.49E+04 | 4.30E+04 | 1.64E+04 | 4.51E+03 | 1.68E+05 | 7.34E+04 | 1.27E+05 | 1.02E+06 | 3.34E+06 | 4.06E+05 |
| 3 | 1,6,10-Dodecatrien-3-ol, 3,7,11-trimethyl-, (E)- | Terpenes | 1.00E+00 | 1.00E+00 | 1.96E+03 | 1.00E+00 | 8.27E+03 | 1.57E+03 | 1.25E+04 | 7.86E+03 | 1.02E+04 | 9.34E+04 | 5.08E+05 | 4.72E+04 |
| 3 | .beta.-Bisabolene | Terpenes | 9.18E+03 | 9.21E+03 | 6.88E+03 | 1.24E+04 | 4.99E+05 | 8.28E+04 | 9.60E+04 | 5.89E+04 | 1.02E+06 | 1.31E+06 | 1.59E+06 | 4.56E+05 |
| 3 | .alpha.-Farnesene | Terpenes | 1.07E+04 | 8.67E+05 | 1.83E+06 | 4.33E+05 | 9.97E+05 | 1.44E+05 | 6.07E+05 | 4.27E+05 | 4.42E+05 | 7.61E+05 | 2.29E+06 | 2.80E+05 |
| 3 | .alpha.-Terpinyl acetate | Ester | 3.10E+03 | 2.33E+04 | 1.53E+04 | 5.54E+03 | 1.75E+04 | 1.83E+04 | 2.69E+04 | 7.86E+04 | 6.04E+04 | 7.77E+04 | 7.55E+04 | 1.61E+04 |
| 3 | Geranyl acetate | Ester | 3.29E+04 | 2.49E+06 | 7.44E+05 | 8.10E+05 | 6.66E+04 | 5.04E+04 | 3.89E+06 | 3.78E+06 | 7.54E+04 | 5.88E+06 | 1.26E+07 | 3.29E+06 |
| 3 | (E)-1-Methyl-4-(6-methylhept-5-en-2-ylidene)cyclohex-1-ene | Terpenes | 1.52E+03 | 2.35E+04 | 6.33E+04 | 1.40E+04 | 1.18E+04 | 1.36E+03 | 2.78E+04 | 1.73E+04 | 1.00E+00 | 1.47E+05 | 6.96E+05 | 4.22E+04 |
| 3 | 1,6,10-Dodecatrien-3-ol, 3,7,11-trimethyl- | Terpenes | 1.00E+00 | 1.00E+00 | 1.96E+03 | 1.00E+00 | 8.27E+03 | 1.57E+03 | 1.25E+04 | 7.86E+03 | 1.02E+04 | 9.34E+04 | 5.08E+05 | 4.72E+04 |
| 3 | 5,9-Undecadien-2-ol, 6,10-dimethyl- | Alcohol | 3.07E+03 | 1.85E+03 | 1.00E+00 | 1.00E+00 | 6.40E+04 | 4.85E+03 | 4.05E+03 | 1.07E+03 | 8.63E+04 | 1.19E+05 | 2.28E+05 | 1.21E+05 |
| 3 | cis-.alpha.-Bergamotene | Terpenes | 6.30E+03 | 5.15E+03 | 1.10E+04 | 4.87E+04 | 4.36E+05 | 8.66E+04 | 1.22E+05 | 8.90E+04 | 1.28E+06 | 3.94E+06 | 2.02E+06 | 1.56E+06 |
| 3 | Benzene, (1-methoxypropyl)- | Aromatics | 1.59E+05 | 1.11E+05 | 4.53E+04 | 1.21E+05 | 1.46E+05 | 9.79E+04 | 1.47E+05 | 1.19E+05 | 1.53E+04 | 1.54E+05 | 2.81E+05 | 1.96E+05 |
| 3 | 2,6,10-Dodecatrienal, 3,7,11-trimethyl- | Terpenes | 1.00E+00 | 1.00E+00 | 1.00E+00 | 1.00E+00 | 3.98E+03 | 4.37E+02 | 1.00E+00 | 1.00E+00 | 1.08E+04 | 5.05E+04 | 2.65E+05 | 2.30E+04 |
| 3 | (1S,5S,6R)-6-Methyl-2-methylene-6-(4-methylpent-3-en-1-yl)bicyclo[3.1.1]heptane | Terpenes | 1.00E+00 | 2.96E+03 | 7.52E+03 | 3.54E+03 | 9.39E+04 | 1.31E+04 | 3.65E+03 | 2.56E+03 | 7.75E+04 | 4.13E+05 | 2.00E+05 | 1.13E+05 |
| 3 | Bicyclo[3.1.1]heptane, 6-methyl-2-methylene-6-(4-methyl-3-pentenyl)-, [1R-(1.alpha.,5.alpha.,6.beta.)]- | Terpenes | 8.97E+03 | 3.85E+04 | 7.78E+04 | 2.28E+04 | 1.00E+00 | 1.00E+00 | 4.50E+04 | 3.04E+04 | 1.13E+04 | 2.33E+05 | 1.18E+06 | 7.94E+04 |
| 3 | Tetrahydro-4,7,8-trimethyl-1,5-benzodiazepin-2-one | Ketone | 2.76E+03 | 2.68E+03 | 1.85E+03 | 4.00E+03 | 1.52E+05 | 2.54E+04 | 2.88E+04 | 1.72E+04 | 3.91E+05 | 4.91E+05 | 5.70E+05 | 1.62E+05 |
| 3 | Isolongifolene, 4,5,9,10-dehydro- | Phenol | 3.22E+03 | 7.12E+03 | 1.33E+04 | 5.30E+03 | 5.27E+04 | 6.31E+03 | 6.42E+03 | 4.09E+03 | 1.25E+05 | 1.42E+05 | 1.98E+05 | 6.56E+04 |
| 3 | 1,3,6,10-Dodecatetraene, 3,7,11-trimethyl-, (Z,E)- | Terpenes | 1.07E+04 | 8.67E+05 | 1.83E+06 | 4.33E+05 | 9.90E+05 | 1.43E+05 | 6.07E+05 | 4.26E+05 | 4.16E+05 | 7.18E+05 | 2.31E+06 | 2.80E+05 |
| 3 | 3-Cyclohexene-1-methanol, .alpha.,4-dimethyl-.alpha.-(4-methyl-3-pentenyl)-, [R-(R*,R*)]- | Terpenes | 1.00E+00 | 1.57E+03 | 1.00E+00 | 1.28E+03 | 2.05E+04 | 1.80E+03 | 1.03E+04 | 5.93E+03 | 2.09E+04 | 8.74E+04 | 4.15E+05 | 3.85E+04 |
| 3 | Cyclohexene, 4-[(1E)-1,5-dimethyl-1,4-hexadien-1-yl]-1-methyl- | Terpenes | 4.13E+03 | 4.40E+03 | 3.80E+03 | 2.14E+03 | 1.88E+05 | 2.17E+04 | 2.92E+04 | 7.97E+03 | 1.50E+05 | 1.99E+05 | 7.95E+05 | 1.08E+05 |
| 3 | Piperitenone oxide | Ketone | 1.00E+00 | 1.00E+00 | 1.27E+03 | 1.00E+00 | 1.30E+04 | 1.24E+03 | 1.77E+03 | 3.35E+03 | 5.75E+03 | 1.14E+04 | 3.10E+04 | 2.86E+03 |
| 4 | α-thujene | Terpenes | 6.04E+03 | 1.09E+05 | 4.15E+04 | 4.44E+04 | 1.22E+04 | 1.06E+04 | 2.86E+05 | 2.09E+05 | 1.77E+04 | 1.47E+05 | 2.27E+05 | 1.72E+05 |
| 4 | 3-Carene | Terpenes | 1.16E+06 | 9.39E+06 | 5.13E+06 | 5.63E+06 | 1.79E+06 | 1.80E+06 | 1.99E+07 | 1.20E+07 | 3.26E+06 | 1.24E+07 | 1.56E+07 | 1.14E+07 |
| 4 | .gamma.-Terpinene | Terpenes | 1.29E+05 | 1.70E+06 | 7.36E+05 | 7.61E+05 | 2.49E+05 | 1.83E+05 | 3.95E+06 | 2.85E+06 | 3.19E+05 | 2.03E+06 | 2.93E+06 | 1.73E+06 |
| 4 | Linalool | Terpenes | 2.22E+06 | 3.42E+06 | 1.52E+06 | 1.67E+06 | 5.55E+06 | 2.65E+06 | 8.45E+06 | 5.33E+06 | 4.12E+06 | 4.71E+06 | 6.24E+06 | 4.74E+06 |
| 4 | Geraniol | Terpenes | 1.93E+06 | 2.84E+07 | 1.28E+07 | 1.50E+07 | 1.83E+06 | 3.05E+06 | 6.57E+07 | 3.65E+07 | 4.60E+06 | 4.07E+07 | 5.45E+07 | 4.50E+07 |
| 4 | .alpha.-Phellandrene 1 | Terpenes | 2.90E+05 | 4.19E+06 | 1.81E+06 | 1.90E+06 | 5.64E+05 | 4.33E+05 | 9.84E+06 | 6.96E+06 | 7.29E+05 | 5.26E+06 | 7.64E+06 | 4.60E+06 |
| 4 | trans-Geranic acid methyl ester | Ester | 7.24E+02 | 5.97E+03 | 4.65E+03 | 2.35E+03 | 7.94E+02 | 9.92E+02 | 2.90E+04 | 1.39E+04 | 1.58E+03 | 3.68E+04 | 3.71E+04 | 9.25E+03 |
| 4 | Bicyclo[3.1.1]heptane, 6,6-dimethyl-2-methylene-, (1S)- | Terpenes | 2.93E+06 | 1.97E+07 | 1.16E+07 | 1.26E+07 | 4.17E+06 | 4.41E+06 | 3.68E+07 | 2.47E+07 | 7.46E+06 | 2.50E+07 | 3.02E+07 | 2.29E+07 |
| 4 | Benzene, 1,4-diethyl- | Aromatics | 3.83E+03 | 5.31E+04 | 2.28E+04 | 2.37E+04 | 5.71E+03 | 6.99E+03 | 2.24E+05 | 7.11E+04 | 1.23E+04 | 1.02E+05 | 1.42E+05 | 9.07E+04 |
| 4 | Bicyclo[3.1.0]hex-2-ene, 4-methyl-1-(1-methylethyl)- | Olefin | 3.68E+03 | 4.23E+04 | 2.29E+04 | 2.22E+04 | 5.60E+03 | 6.62E+03 | 1.41E+05 | 9.03E+04 | 6.72E+03 | 5.95E+04 | 8.85E+04 | 6.48E+04 |
| 4 | Benzene, 1-methyl-3-(1-methylethyl)- | Aromatics | 6.58E+05 | 2.49E+06 | 1.11E+06 | 1.40E+06 | 1.22E+06 | 4.89E+05 | 4.96E+06 | 3.60E+06 | 8.07E+05 | 2.55E+06 | 3.42E+06 | 2.48E+06 |
| 4 | (+)-4-Carene | Terpenes | 2.86E+05 | 2.35E+06 | 1.08E+06 | 1.13E+06 | 4.11E+05 | 2.79E+05 | 4.96E+06 | 3.92E+06 | 4.67E+05 | 2.79E+06 | 4.02E+06 | 2.47E+06 |
| 4 | 2,4,6-Octatriene, 2,6-dimethyl- | Terpenes | 1.68E+03 | 1.04E+04 | 5.35E+03 | 6.36E+03 | 1.97E+03 | 2.43E+03 | 2.79E+04 | 1.27E+04 | 3.76E+03 | 1.53E+04 | 2.12E+04 | 1.62E+04 |
| 4 | Bicyclo[2.2.1]hept-2-ene, 1,7,7-trimethyl- | Terpenes | 4.63E+03 | 8.78E+04 | 4.48E+04 | 4.00E+04 | 4.49E+04 | 1.48E+04 | 2.36E+05 | 1.80E+05 | 1.10E+04 | 1.16E+05 | 1.67E+05 | 9.42E+04 |
| 4 | Cyclohexene, 4-methyl-1-(1-methylethenyl)- | Olefin | 2.37E+04 | 5.89E+04 | 3.24E+04 | 5.21E+04 | 4.99E+04 | 1.18E+04 | 1.76E+05 | 1.55E+05 | 3.91E+04 | 9.84E+04 | 1.80E+05 | 9.92E+04 |
| 4 | 2-Amino-2-methyl-but-3-enoic acid | Acid | 9.63E+04 | 6.63E+05 | 3.86E+05 | 4.26E+05 | 1.37E+05 | 1.46E+05 | 1.30E+06 | 8.49E+05 | 2.49E+05 | 8.59E+05 | 1.04E+06 | 7.84E+05 |
| 4 | 2,4,6-Octatriene, 3,4-dimethyl- | Olefin | 5.72E+04 | 6.63E+05 | 2.73E+05 | 3.16E+05 | 1.19E+05 | 9.14E+04 | 2.02E+06 | 9.28E+05 | 1.95E+05 | 1.11E+06 | 1.47E+06 | 9.84E+05 |
| 4 | Phenol, m-tert-butyl- | Phenol | 2.85E+04 | 2.74E+05 | 1.10E+05 | 1.28E+05 | 5.87E+04 | 3.44E+04 | 1.04E+06 | 4.17E+05 | 7.73E+04 | 4.88E+05 | 6.92E+05 | 4.32E+05 |
| 4 | Benzene, n-butyl- | Aromatics | 2.60E+05 | 8.13E+05 | 3.63E+05 | 4.72E+05 | 4.50E+05 | 1.56E+05 | 1.64E+06 | 1.22E+06 | 2.63E+05 | 8.35E+05 | 1.12E+06 | 8.12E+05 |
| 4 | p-Mentha-1,5,8-triene | Terpenes | 5.86E+04 | 1.00E+00 | 1.20E+05 | 3.18E+05 | 7.90E+04 | 7.90E+04 | 9.38E+05 | 4.96E+05 | 1.80E+05 | 5.39E+05 | 6.91E+05 | 6.26E+05 |
| 4 | Phenol, 4-propyl- | Phenol | 1.15E+04 | 1.34E+05 | 5.27E+04 | 5.86E+04 | 1.85E+04 | 1.60E+04 | 5.35E+05 | 1.93E+05 | 3.76E+04 | 2.41E+05 | 3.38E+05 | 2.04E+05 |
| 4 | Phenol, 4-(1-methylpropyl)- | Phenol | 3.03E+05 | 2.04E+06 | 1.47E+06 | 1.51E+06 | 4.74E+05 | 4.85E+05 | 8.19E+06 | 2.47E+06 | 9.82E+05 | 5.24E+06 | 6.18E+06 | 4.50E+06 |
| 4 | Cyclohexyl methyl methylphosphonate | Ester | 2.09E+04 | 1.80E+05 | 9.40E+04 | 6.87E+04 | 1.53E+04 | 1.61E+04 | 3.68E+05 | 1.87E+05 | 3.07E+04 | 2.58E+05 | 3.30E+05 | 2.24E+05 |
| 4 | 1,5-Heptadiene, 2,6-dimethyl- | Olefin | 6.63E+03 | 4.86E+04 | 2.60E+04 | 3.17E+04 | 8.48E+03 | 5.65E+03 | 1.47E+05 | 8.14E+04 | 2.62E+04 | 7.23E+04 | 1.03E+05 | 7.46E+04 |
| 4 | 2,6-Dimethyl-2-trans-6-octadiene | Terpenes | 1.12E+04 | 4.70E+04 | 2.15E+04 | 1.46E+04 | 9.03E+03 | 4.22E+03 | 2.13E+05 | 7.99E+04 | 2.79E+04 | 9.39E+04 | 1.15E+05 | 6.81E+04 |
| 4 | 1,4-Methano-1H-Cyclopropa[d]pyridazine, 4,4a,5,5a-tetrahydro-6,6-dimethyl-, (1.alpha.,4.alpha.,4a.alpha.,5a.alpha.)- | Heterocyclic compound | 9.85E+05 | 9.61E+06 | 5.14E+06 | 5.33E+06 | 1.56E+06 | 1.34E+06 | 1.81E+07 | 1.43E+07 | 2.07E+06 | 1.12E+07 | 1.51E+07 | 1.01E+07 |
| 4 | Benzene, 2-ethyl-1,4-dimethyl- | Aromatics | 3.19E+04 | 4.61E+05 | 2.07E+05 | 2.17E+05 | 4.63E+04 | 4.91E+04 | 1.39E+06 | 6.06E+05 | 9.79E+04 | 8.21E+05 | 9.93E+05 | 6.69E+05 |
| 4 | 5-propan-2-ylbicyclo[3.1.0]hex-2-ene-2-carbaldehyde | Aldehyde | 6.72E+04 | 5.10E+05 | 2.33E+05 | 2.66E+05 | 1.34E+05 | 8.69E+04 | 1.43E+06 | 8.03E+05 | 1.86E+05 | 7.85E+05 | 1.06E+06 | 7.17E+05 |
| 4 | Phosphinic acid, diethyl-, methyl ester | Ester | 5.60E+04 | 3.82E+05 | 1.59E+05 | 1.81E+05 | 8.71E+04 | 7.39E+04 | 1.38E+06 | 5.98E+05 | 1.21E+05 | 6.76E+05 | 9.26E+05 | 5.65E+05 |
| 4 | 2-Methylindene | Aromatics | 3.88E+03 | 1.15E+04 | 5.26E+03 | 7.42E+03 | 3.32E+03 | 3.11E+03 | 2.85E+04 | 1.85E+04 | 5.50E+03 | 1.39E+04 | 1.72E+04 | 1.44E+04 |
| 5 | Styrene | Aromatics | 6.11E+04 | 3.32E+05 | 3.89E+05 | 2.65E+05 | 1.44E+05 | 7.06E+04 | 1.77E+05 | 1.56E+05 | 3.42E+04 | 4.09E+04 | 2.60E+04 | 3.74E+04 |
| 5 | Phenylethyl Alcohol | Alcohol | 2.40E+07 | 1.68E+08 | 1.25E+08 | 8.49E+07 | 1.39E+07 | 1.88E+07 | 8.64E+07 | 7.42E+07 | 1.57E+06 | 3.87E+05 | 1.00E+00 | 1.18E+06 |
| 5 | Benzeneacetaldehyde | Aldehyde | 7.28E+05 | 1.15E+06 | 1.04E+06 | 1.20E+06 | 7.90E+05 | 1.78E+05 | 5.77E+05 | 9.05E+05 | 1.39E+05 | 8.24E+04 | 1.83E+05 | 2.72E+05 |
| 5 | 3-Octanone | Ketone | 6.15E+05 | 5.03E+05 | 3.13E+05 | 4.17E+05 | 7.16E+04 | 4.15E+04 | 5.46E+03 | 1.12E+04 | 6.45E+05 | 9.32E+04 | 3.03E+05 | 1.68E+05 |
| 5 | Pentadecane | Alkanes | 3.71E+04 | 6.13E+05 | 6.73E+05 | 1.90E+05 | 6.38E+04 | 6.27E+04 | 2.24E+05 | 1.94E+05 | 5.24E+05 | 3.84E+05 | 3.24E+05 | 4.63E+04 |
| 5 | Hexadecane | Alkanes | 1.00E+00 | 5.32E+05 | 3.88E+05 | 1.89E+05 | 3.27E+03 | 3.37E+03 | 4.18E+04 | 3.20E+04 | 1.47E+04 | 4.97E+04 | 1.08E+05 | 2.39E+04 |
| 5 | Heptadecane | Alkanes | 1.78E+04 | 1.73E+05 | 2.15E+05 | 1.12E+05 | 1.83E+04 | 2.71E+04 | 6.56E+04 | 9.25E+04 | 5.93E+04 | 1.08E+05 | 1.92E+05 | 4.04E+04 |
| 5 | Heneicosane | Alkanes | 3.18E+03 | 1.00E+06 | 7.19E+05 | 4.38E+05 | 2.26E+04 | 4.14E+04 | 1.02E+05 | 8.28E+04 | 5.05E+04 | 1.02E+05 | 1.91E+05 | 1.15E+05 |
| 5 | p-Xylene | Aromatics | 1.91E+04 | 3.21E+04 | 7.07E+04 | 5.44E+04 | 4.45E+04 | 3.53E+04 | 2.15E+04 | 3.07E+04 | 2.46E+04 | 2.60E+04 | 1.29E+04 | 2.22E+04 |
| 5 | 1-Pentanone, 1-(4-methylphenyl)- | Ketone | 3.78E+03 | 2.94E+04 | 1.00E+00 | 1.00E+00 | 2.48E+03 | 1.00E+00 | 8.34E+03 | 1.09E+04 | 6.75E+03 | 3.88E+03 | 8.51E+03 | 1.53E+04 |
| 5 | Benzene, 1,2-dichloro- | Aromatics | 1.72E+04 | 1.89E+04 | 4.47E+04 | 2.98E+04 | 7.93E+03 | 6.04E+03 | 6.51E+03 | 5.61E+03 | 7.83E+03 | 4.74E+03 | 4.51E+03 | 7.57E+03 |
| 5 | Methanesulfinyl fluoride | Other | 5.27E+05 | 1.80E+06 | 2.02E+06 | 1.34E+06 | 5.60E+05 | 4.17E+05 | 3.12E+05 | 1.47E+05 | 6.72E+05 | 3.52E+05 | 2.59E+05 | 9.62E+04 |
| 5 | 4-Penten-1-ol, propanoate | Ester | 2.46E+05 | 1.34E+05 | 5.80E+04 | 7.11E+04 | 9.97E+03 | 1.01E+04 | 9.83E+03 | 4.29E+04 | 1.23E+05 | 1.71E+04 | 4.39E+04 | 3.56E+04 |
| 5 | Butanoic acid, 2-methyl-, phenylmethyl ester | Ester | 1.62E+05 | 7.27E+03 | 9.78E+03 | 8.37E+03 | 5.09E+04 | 1.00E+00 | 1.00E+00 | 1.00E+00 | 1.00E+00 | 1.00E+00 | 1.00E+00 | 1.00E+00 |
| 5 | Butanoic acid, 2-methyl-, 2-phenylethyl ester | Ester | 1.07E+06 | 7.60E+04 | 3.97E+05 | 1.83E+05 | 8.70E+04 | 1.00E+00 | 1.96E+04 | 1.20E+05 | 1.00E+00 | 1.00E+00 | 1.00E+00 | 1.00E+00 |
| 6 | Citronellal | Terpenes | 4.75E+04 | 1.86E+05 | 1.60E+05 | 3.14E+05 | 1.24E+05 | 5.35E+04 | 6.81E+05 | 1.03E+06 | 2.40E+05 | 6.68E+05 | 8.21E+05 | 4.45E+05 |
| 6 | Citronellol | Terpenes | 2.00E+04 | 2.40E+06 | 1.87E+06 | 6.65E+05 | 2.34E+04 | 9.04E+04 | 4.96E+06 | 4.90E+06 | 2.88E+04 | 2.93E+06 | 2.26E+06 | 1.73E+06 |
| 6 | Terpinen-4-ol | Terpenes | 1.22E+04 | 4.32E+04 | 1.63E+04 | 1.96E+04 | 3.79E+04 | 1.03E+04 | 1.02E+05 | 1.01E+05 | 1.32E+04 | 5.71E+04 | 7.90E+04 | 4.64E+04 |
| 6 | 2,6-Octadien-1-ol, 3,7-dimethyl-, (Z)- | Alcohol | 6.99E+03 | 3.21E+05 | 1.59E+05 | 1.66E+05 | 3.62E+03 | 8.32E+03 | 5.73E+05 | 6.97E+05 | 8.38E+03 | 3.11E+05 | 6.23E+05 | 3.36E+05 |
| 6 | Benzene, 1-ethenyl-4-methoxy- | Aromatics | 6.15E+03 | 1.00E+00 | 1.00E+00 | 1.71E+04 | 9.46E+03 | 4.06E+03 | 4.41E+04 | 4.45E+04 | 5.46E+03 | 2.51E+04 | 3.93E+04 | 2.94E+04 |
| 6 | Heptanal | Aldehyde | 7.15E+03 | 2.03E+05 | 1.02E+05 | 9.57E+04 | 1.01E+04 | 2.47E+03 | 7.86E+05 | 6.15E+05 | 1.98E+04 | 7.80E+04 | 6.25E+04 | 1.53E+05 |
| 6 | 2-Undecanone | Ketone | 1.44E+03 | 1.32E+05 | 3.04E+05 | 2.08E+05 | 3.52E+03 | 1.80E+04 | 7.43E+05 | 6.58E+05 | 3.56E+03 | 1.77E+05 | 4.90E+04 | 3.59E+04 |
| 6 | 6-Octen-1-ol, 3,7-dimethyl-, acetate | Ester | 3.20E+03 | 1.53E+06 | 1.91E+06 | 2.87E+05 | 4.18E+03 | 1.28E+04 | 2.40E+06 | 5.34E+06 | 6.21E+04 | 3.16E+06 | 3.41E+06 | 5.82E+05 |
| 6 | Benzene, 1-methyl-3-(1-methylethenyl)- | Aromatics | 2.31E+05 | 4.98E+05 | 2.20E+05 | 2.87E+05 | 4.31E+05 | 1.20E+05 | 9.14E+05 | 7.42E+05 | 2.09E+05 | 4.52E+05 | 5.92E+05 | 4.42E+05 |
| 6 | 2,7-Nonadien-5-one, 4,6-dimethyl- | Ketone | 2.94E+05 | 2.97E+07 | 2.07E+07 | 1.20E+07 | 4.01E+05 | 1.28E+06 | 5.32E+07 | 5.69E+07 | 4.59E+05 | 3.40E+07 | 3.88E+07 | 2.53E+07 |
| 6 | trans-8-Ethyl-bicyclo[4.3.0]non-3-ene | Olefin | 3.62E+04 | 3.33E+04 | 1.39E+04 | 2.06E+04 | 3.09E+04 | 2.80E+04 | 9.70E+04 | 1.06E+05 | 1.44E+04 | 5.98E+04 | 7.86E+04 | 5.30E+04 |
| 6 | 3-Cyclohexen-1-ol, 4-methyl-1-(1-methylethyl)-, (R)- | Terpenes | 1.22E+04 | 4.31E+04 | 1.57E+04 | 2.08E+04 | 3.71E+04 | 1.03E+04 | 1.02E+05 | 1.01E+05 | 1.30E+04 | 5.69E+04 | 7.90E+04 | 4.55E+04 |
| 6 | 6-Octen-1-ol, 3,7-dimethyl-, (R)- | Alcohol | 1.44E+05 | 1.25E+07 | 1.00E+07 | 4.06E+06 | 1.64E+05 | 5.96E+05 | 2.22E+07 | 2.25E+07 | 2.07E+05 | 1.45E+07 | 1.19E+07 | 9.36E+06 |
| 6 | 2-Tridecanone | Ketone | 1.00E+00 | 1.21E+05 | 3.11E+05 | 2.68E+05 | 4.08E+03 | 1.02E+04 | 1.57E+06 | 2.54E+06 | 6.95E+03 | 6.89E+05 | 8.71E+05 | 2.58E+05 |
| 7 | (2S,4R)-4-Methyl-2-(2-methylprop-1-en-1-yl)tetrahydro-2H-pyran | Terpenes | 2.93E+03 | 1.27E+06 | 2.39E+06 | 4.12E+06 | 2.38E+04 | 4.68E+04 | 3.12E+06 | 1.87E+07 | 1.35E+04 | 1.30E+06 | 7.51E+06 | 4.72E+06 |
| 7 | 2H-Pyran, 3,6-dihydro-4-methyl-2-(2-methyl-1-propenyl)- | Terpenes | 4.98E+04 | 4.32E+05 | 3.45E+05 | 1.34E+06 | 3.04E+04 | 2.96E+04 | 4.68E+05 | 2.93E+06 | 1.28E+05 | 2.35E+05 | 1.95E+06 | 1.26E+06 |
| 7 | Benzene, 1,2,3-trimethoxy-5-(2-propenyl)- | Aromatics | 1.00E+00 | 3.20E+04 | 1.76E+05 | 7.48E+04 | 1.00E+00 | 1.00E+00 | 5.25E+04 | 2.58E+05 | 4.54E+03 | 1.38E+05 | 2.29E+05 | 4.74E+04 |
| 7 | Methyleugenol | Phenol | 3.48E+03 | 3.40E+06 | 1.22E+07 | 1.80E+06 | 1.24E+04 | 2.30E+04 | 3.91E+06 | 1.52E+07 | 7.37E+05 | 3.81E+06 | 7.50E+06 | 1.67E+06 |
| 7 | Acetic acid, 2-phenylethyl ester | Ester | 1.01E+06 | 1.28E+06 | 1.67E+06 | 1.32E+06 | 2.44E+05 | 7.93E+05 | 1.64E+06 | 3.85E+06 | 2.26E+04 | 3.44E+04 | 4.83E+04 | 4.64E+04 |
| 7 | Undecane, 2,5-dimethyl- | Alkanes | 2.34E+04 | 2.51E+04 | 2.36E+04 | 2.38E+04 | 1.97E+04 | 3.54E+04 | 2.46E+04 | 2.94E+04 | 2.34E+04 | 1.58E+04 | 2.73E+04 | 1.74E+04 |
| 7 | Ethyl 4-(ethyloxy)-2-oxobut-3-enoate | Ester | 7.07E+03 | 3.95E+04 | 6.80E+04 | 1.21E+05 | 1.40E+03 | 2.43E+03 | 1.26E+05 | 9.09E+05 | 4.23E+03 | 3.25E+04 | 2.46E+05 | 1.21E+05 |
| 7 | 2H-Pyran, tetrahydro-4-methyl-2-(2-methyl-1-propenyl)- | Terpenes | 6.44E+03 | 2.49E+06 | 4.18E+06 | 8.19E+06 | 5.66E+04 | 1.14E+05 | 7.99E+06 | 3.71E+07 | 3.69E+04 | 3.20E+06 | 1.45E+07 | 9.45E+06 |
| 8 | Naphthalene, 1,2,3,4,4a,5,6,8a-octahydro-7-methyl-4-methylene-1-(1-methylethyl)-, (1.alpha.,4a.beta.,8a.alpha.)- | Terpenes | 1.19E+04 | 7.65E+04 | 3.41E+04 | 1.02E+05 | 1.00E+06 | 2.25E+05 | 4.33E+05 | 2.34E+05 | 5.33E+05 | 3.28E+05 | 4.01E+05 | 1.28E+05 |
| 8 | Cubenene | Terpenes | 1.93E+04 | 7.49E+03 | 1.14E+04 | 5.82E+03 | 6.65E+05 | 7.97E+04 | 6.71E+04 | 5.22E+04 | 1.73E+05 | 4.74E+04 | 6.63E+04 | 2.61E+04 |
| 8 | 1-Hexanol | Alcohol | 9.19E+05 | 5.77E+05 | 6.69E+05 | 1.67E+06 | 1.95E+06 | 2.35E+05 | 6.47E+05 | 1.37E+06 | 8.21E+05 | 5.16E+05 | 4.94E+05 | 8.62E+05 |
| 8 | Benzaldehyde | Aldehyde | 2.34E+05 | 4.29E+05 | 3.04E+05 | 3.53E+05 | 6.19E+05 | 1.69E+05 | 2.39E+05 | 2.16E+05 | 3.86E+05 | 1.84E+05 | 1.59E+05 | 1.75E+05 |
| 8 | Decanal | Aldehyde | 1.77E+04 | 5.73E+03 | 5.99E+03 | 6.56E+03 | 1.60E+04 | 6.06E+03 | 4.49E+03 | 6.95E+03 | 1.48E+04 | 4.82E+03 | 8.88E+03 | 1.01E+04 |
| 8 | Dill ether | Heterocyclic compound | 5.82E+03 | 4.70E+03 | 4.46E+03 | 9.74E+03 | 7.45E+03 | 4.32E+03 | 4.79E+03 | 6.89E+03 | 8.79E+03 | 2.41E+03 | 5.19E+03 | 4.72E+03 |
| 8 | Acetic acid, hexyl ester | Ester | 4.32E+04 | 1.58E+03 | 5.17E+03 | 1.09E+04 | 8.44E+04 | 1.36E+04 | 1.46E+04 | 3.28E+04 | 2.07E+04 | 6.09E+04 | 1.73E+04 | 2.30E+04 |
| 8 | (3aR,4R,7R)-1,4,9,9-Tetramethyl-3,4,5,6,7,8-hexahydro-2H-3a,7-methanoazulen-2-one | Terpenes | 3.44E+03 | 2.16E+03 | 1.00E+00 | 1.00E+00 | 3.84E+05 | 2.45E+04 | 2.32E+04 | 1.16E+04 | 6.24E+04 | 2.93E+04 | 3.15E+04 | 7.67E+03 |
| 8 | (+)-alpha-Pinene | Terpenes | 9.79E+03 | 5.53E+04 | 3.40E+04 | 4.35E+04 | 7.97E+05 | 4.87E+05 | 9.27E+05 | 5.21E+05 | 2.17E+05 | 6.52E+05 | 1.86E+05 | 7.16E+04 |
| 8 | Tetradecane, 4-methyl- | Alkanes | 1.01E+04 | 1.24E+04 | 1.25E+04 | 1.05E+04 | 3.10E+04 | 1.63E+04 | 1.88E+04 | 2.22E+04 | 1.78E+04 | 1.66E+04 | 2.46E+04 | 1.49E+04 |
| 8 | (1S,4S,4aS)-1-Isopropyl-4,7-dimethyl-1,2,3,4,4a,5-hexahydronaphthalene | Terpenes | 1.37E+05 | 4.90E+05 | 2.04E+05 | 6.00E+05 | 2.04E+06 | 3.95E+05 | 8.04E+05 | 3.61E+05 | 9.98E+05 | 4.73E+05 | 5.37E+05 | 1.34E+05 |
| 8 | .alpha.-Dehydro-ar-himachalene | Terpenes | 9.82E+03 | 4.85E+03 | 2.45E+03 | 6.76E+03 | 1.60E+05 | 1.89E+04 | 8.65E+03 | 7.61E+03 | 1.05E+05 | 7.44E+03 | 9.90E+03 | 4.59E+03 |
| 8 | 1,1,7,7a-Tetramethyl-1a,2,6,7,7a,7b-hexahydro-1H-cyclopropa[a]naphthalene | Terpenes | 2.45E+04 | 1.17E+05 | 5.85E+04 | 1.79E+05 | 1.06E+06 | 1.56E+05 | 1.65E+05 | 9.66E+04 | 3.99E+05 | 9.50E+04 | 7.91E+04 | 3.33E+04 |
| 8 | 2H-Tetrazole, 2-methyl- | Heterocyclic compound | 9.15E+05 | 5.76E+05 | 6.69E+05 | 1.67E+06 | 1.95E+06 | 2.33E+05 | 6.47E+05 | 1.36E+06 | 8.18E+05 | 5.16E+05 | 4.94E+05 | 8.62E+05 |
| 8 | (-)-cis-Isopiperitenol | Terpenes | 9.81E+03 | 7.05E+03 | 1.00E+00 | 1.00E+00 | 4.92E+04 | 8.95E+03 | 2.15E+04 | 2.48E+04 | 1.12E+04 | 1.54E+04 | 1.27E+04 | 1.03E+04 |
| 8 | Tricyclo[4.4.0.02,7]decane, 1-methyl-3-methylene-8-(1-methylethyl)-, stereoisomer | Terpenes | 6.26E+04 | 5.57E+04 | 2.24E+04 | 3.26E+04 | 2.82E+06 | 5.18E+05 | 8.50E+05 | 6.51E+05 | 1.11E+06 | 7.45E+05 | 8.77E+05 | 3.17E+05 |
| 8 | Pyrimidine, 4-(2-hydroxy-5-methoxyphenyl)- | Heterocyclic compound | 3.30E+03 | 6.60E+03 | 2.93E+03 | 8.65E+03 | 1.36E+05 | 1.67E+04 | 1.30E+04 | 8.99E+03 | 3.13E+04 | 1.54E+04 | 7.00E+03 | 3.86E+03 |
| 8 | 8-Isopropyl-1-methyltricyclo[4.4.0.02,7]dec-3-ene-3-carbaldehyde | Aldehyde | 2.95E+04 | 1.58E+04 | 1.04E+03 | 1.43E+03 | 3.04E+06 | 2.84E+05 | 3.66E+05 | 1.06E+05 | 9.56E+03 | 1.41E+04 | 1.60E+04 | 8.88E+03 |
| 8 | Bicyclo[3.1.0]hex-2-ene, 4-methylene-1-(1-methylethyl)- | Terpenes | 1.96E+04 | 5.91E+04 | 3.23E+04 | 3.85E+04 | 5.67E+05 | 1.08E+05 | 1.83E+05 | 1.81E+05 | 1.32E+05 | 1.56E+05 | 1.13E+05 | 8.15E+04 |
| 8 | (1R,5S)-1,8-Dimethyl-4-(propan-2-ylidene)spiro[4.5]dec-7-ene | Terpenes | 1.41E+03 | 2.57E+03 | 1.00E+00 | 3.36E+03 | 8.24E+04 | 8.83E+03 | 1.21E+04 | 8.10E+03 | 3.19E+04 | 1.05E+04 | 1.26E+04 | 3.27E+03 |
| 8 | Benzofuran, 7-cyclohexyl-2,3-dihydro-2-methyl- | Heterocyclic compound | 1.19E+04 | 2.76E+03 | 1.00E+00 | 1.00E+00 | 7.50E+05 | 6.95E+04 | 4.90E+04 | 1.97E+04 | 3.12E+03 | 2.35E+03 | 3.39E+03 | 2.44E+03 |
| 9 | (-)-.beta.-Bourbonene | Terpenes | 1.65E+04 | 5.15E+03 | 3.87E+03 | 3.37E+03 | 2.89E+04 | 8.27E+03 | 1.17E+04 | 1.32E+04 | 1.28E+05 | 4.75E+05 | 1.47E+05 | 7.80E+04 |
| 9 | Naphthalene, 1,2,3,5,6,7,8,8a-octahydro-1,8a-dimethyl-7-(1-methylethenyl)-, [1R-(1.alpha.,7.beta.,8a.alpha.)]- | Terpenes | 4.12E+03 | 1.00E+00 | 1.00E+00 | 1.00E+00 | 2.02E+05 | 2.52E+04 | 2.55E+04 | 1.82E+04 | 4.21E+05 | 1.64E+05 | 1.85E+05 | 4.77E+04 |
| 9 | Naphthalene, 1,2,3,5,6,8a-hexahydro-4,7-dimethyl-1-(1-methylethyl)-, (1S-cis)- | Terpenes | 1.32E+04 | 4.32E+03 | 1.77E+03 | 2.50E+03 | 4.07E+05 | 2.87E+04 | 2.97E+04 | 2.35E+04 | 1.13E+06 | 3.85E+05 | 3.21E+05 | 8.18E+04 |
| 9 | Naphthalene, 1,2,3,4-tetrahydro-1,6-dimethyl-4-(1-methylethyl)-, (1S-cis)- | Terpenes | 3.10E+04 | 9.90E+03 | 5.24E+03 | 8.38E+03 | 2.37E+05 | 2.28E+04 | 1.48E+04 | 1.32E+04 | 1.45E+06 | 4.91E+05 | 4.14E+05 | 1.63E+05 |
| 9 | .alpha.-Calacorene | Terpenes | 1.51E+04 | 4.86E+03 | 2.22E+03 | 3.14E+03 | 1.16E+05 | 6.85E+03 | 6.09E+03 | 6.07E+03 | 6.69E+05 | 2.62E+05 | 1.79E+05 | 7.87E+04 |
| 9 | Hexanal | Aldehyde | 2.09E+06 | 5.26E+06 | 2.60E+06 | 1.56E+06 | 3.48E+06 | 5.23E+06 | 4.49E+06 | 1.25E+06 | 9.68E+06 | 5.50E+06 | 2.81E+06 | 6.42E+05 |
| 9 | 2-Hexenal, (E)- | Aldehyde | 2.85E+06 | 3.96E+06 | 2.77E+06 | 2.04E+06 | 4.70E+06 | 5.53E+06 | 3.85E+06 | 1.81E+06 | 8.62E+06 | 4.73E+06 | 3.41E+06 | 1.05E+06 |
| 9 | Tridecane | Alkanes | 2.55E+04 | 1.24E+05 | 1.12E+05 | 4.38E+04 | 3.20E+04 | 3.77E+04 | 1.95E+05 | 5.14E+04 | 6.65E+05 | 5.61E+05 | 1.82E+05 | 5.64E+04 |
| 9 | Methyl salicylate | Ester | 5.24E+05 | 7.17E+04 | 2.68E+04 | 1.17E+05 | 2.03E+06 | 5.08E+05 | 1.80E+05 | 2.21E+05 | 4.61E+06 | 1.97E+06 | 2.58E+06 | 1.97E+06 |
| 9 | .alfa.-Copaene | Terpenes | 1.14E+04 | 4.95E+03 | 4.48E+03 | 1.89E+04 | 1.11E+05 | 1.39E+04 | 5.21E+04 | 1.75E+04 | 3.35E+06 | 2.94E+06 | 1.90E+06 | 9.67E+05 |
| 9 | 3-Methylpenta-1,3-diene-5-ol, (E)- | Alcohol | 7.05E+04 | 8.48E+04 | 4.76E+04 | 3.54E+04 | 1.35E+05 | 1.63E+05 | 9.02E+04 | 4.30E+04 | 3.22E+05 | 1.23E+05 | 7.43E+04 | 2.35E+04 |
| 9 | 6-Isopropyl-1,4-dimethylnaphthalene | Terpenes | 6.87E+03 | 2.09E+03 | 1.95E+03 | 2.38E+03 | 2.51E+04 | 3.80E+03 | 2.85E+03 | 3.02E+03 | 1.35E+05 | 3.91E+04 | 3.10E+04 | 1.58E+04 |
| 9 | Benzene, 1-methyl-4-(1,2,2-trimethylcyclopentyl)-, (R)- | Terpenes | 1.51E+04 | 1.07E+04 | 8.81E+03 | 1.36E+04 | 6.68E+05 | 1.21E+05 | 1.11E+05 | 8.39E+04 | 8.71E+05 | 8.15E+05 | 7.92E+05 | 3.06E+05 |
| 9 | Isolongifolen-5-one | Terpenes | 1.55E+03 | 5.69E+02 | 1.00E+00 | 1.00E+00 | 5.06E+04 | 1.00E+00 | 1.00E+00 | 1.00E+00 | 1.13E+05 | 5.30E+04 | 1.45E+04 | 4.91E+03 |
| 9 | 1-((1R,2R,3R)-2-(3-Isopropylfuran-2-yl)-3-methylcyclopentyl)ethanone | Heterocyclic compound | 8.19E+02 | 1.00E+00 | 1.00E+00 | 3.21E+02 | 1.43E+04 | 4.32E+02 | 7.22E+02 | 8.00E+02 | 9.67E+04 | 1.06E+05 | 1.33E+04 | 1.76E+04 |
| 9 | Nonane, 5-methyl-5-propyl- | Alkanes | 1.15E+05 | 7.88E+03 | 8.55E+03 | 1.13E+04 | 1.06E+05 | 1.94E+05 | 1.00E+00 | 1.00E+00 | 2.87E+05 | 7.19E+03 | 1.13E+04 | 8.64E+03 |
| 9 | isoledene | Terpenes | 1.00E+00 | 1.00E+00 | 1.00E+00 | 1.00E+00 | 1.00E+00 | 1.00E+00 | 1.00E+00 | 1.00E+00 | 5.47E+05 | 1.94E+06 | 6.44E+04 | 9.53E+04 |
| 9 | 1,7-Octadiene, 2-methyl-6-methylene- | Terpenes | 1.66E+06 | 5.54E+05 | 1.00E+06 | 1.35E+06 | 1.53E+06 | 1.60E+06 | 1.23E+06 | 1.21E+06 | 1.70E+06 | 1.55E+06 | 1.68E+06 | 1.60E+06 |
| 9 | Cyclobutanone, 2,2,3-trimethyl- | Ketone | 1.10E+04 | 1.48E+04 | 1.50E+04 | 3.05E+04 | 2.66E+04 | 1.83E+04 | 6.38E+03 | 9.32E+03 | 7.21E+04 | 2.58E+04 | 1.84E+04 | 2.91E+04 |
| 9 | 1,3-Pentanedione, 4,4-dimethyl-1-phenyl- | Ketone | 1.00E+00 | 1.00E+00 | 1.00E+00 | 1.24E+02 | 2.33E+04 | 1.00E+00 | 1.00E+00 | 1.00E+00 | 2.94E+04 | 1.92E+04 | 1.36E+04 | 5.46E+03 |
| 9 | Benzene, (1-methyl-1-propylpentyl)- | Aromatics | 1.00E+00 | 1.00E+00 | 2.20E+03 | 1.82E+03 | 4.03E+04 | 4.82E+03 | 6.61E+03 | 7.63E+03 | 5.27E+04 | 9.66E+04 | 3.21E+04 | 1.31E+04 |
| 9 | l-Alanine, N-(2,3,4-trifluorobenzoyl)-, methyl ester | Ester | 2.79E+03 | 1.00E+00 | 1.00E+00 | 1.00E+00 | 2.02E+04 | 1.00E+00 | 1.00E+00 | 1.00E+00 | 1.05E+05 | 3.43E+04 | 2.44E+04 | 1.26E+04 |
| 9 | Dimethylphosphinic fluoride | Other | 4.45E+05 | 5.31E+05 | 2.55E+05 | 2.02E+05 | 7.56E+05 | 8.63E+05 | 4.64E+05 | 1.98E+05 | 1.95E+06 | 6.09E+05 | 4.73E+05 | 1.51E+05 |
| 9 | 1-(3-Ethoxyphenyl)acetone | Ketone | 4.75E+03 | 2.46E+04 | 1.64E+04 | 4.97E+03 | 3.29E+04 | 3.07E+04 | 2.19E+04 | 2.19E+03 | 4.62E+04 | 2.99E+04 | 1.80E+04 | 3.87E+03 |
| 9 | .beta.-Oplopenone | Terpenes | 1.72E+03 | 1.00E+00 | 1.00E+00 | 1.00E+00 | 1.23E+04 | 1.00E+00 | 1.00E+00 | 1.00E+00 | 7.37E+04 | 4.11E+04 | 5.24E+04 | 2.25E+04 |

Note: sub class represents the metabolite category number with the same trend. They are repeated for three times, and the average value is taken.
